# Ultrafast bisulfite sequencing detection of 5-methylcytosine in DNA and RNA

**DOI:** 10.1038/s41587-023-02034-w

**Published:** 2024-01-02

**Authors:** Qing Dai, Chang Ye, Iryna Irkliyenko, Yiding Wang, Hui-Lung Sun, Yun Gao, Yushuai Liu, Alana Beadell, José Perea, Ajay Goel, Chuan He

**Affiliations:** 1 Department of Chemistry, The University of Chicago, Chicago, IL, USA.; 2 Howard Hughes Medical Institute, The University of Chicago, Chicago, IL, USA.; 3 Committee on Genetics, Genomics & System Biology, The University of Chicago, Chicago, IL, USA.; 4 Institute of Biomedical Research of Salamanca, Salamanca, Spain.; 5 Department of Molecular Diagnostics and Experimental Therapeutics, Beckman Research Institute of City of Hope, Monrovia, CA, USA.; 6 Department of Biochemistry and Molecular Biology, The University of Chicago, Chicago, IL, USA.; 7 Institute for Biophysical Dynamics, The University of Chicago, Chicago, IL, USA.; 8 These authors contributed equally: Qing Dai, Chang Ye, Iryna Irkliyenko, Yiding Wang.

## Abstract

Bisulfite sequencing (BS-seq) to detect 5-methylcytosine (5mC) is limited by lengthy reaction times, severe DNA damage, overestimation of 5mC level and incomplete C-to-U conversion of certain DNA sequences. We present ultrafast BS-seq (UBS-seq), which uses highly concentrated bisulfite reagents and high reaction temperatures to accelerate the bisulfite reaction by ~13-fold, resulting in reduced DNA damage and lower background noise. UBS-seq allows library construction from small amounts of purified genomic DNA, such as from cell-free DNA or directly from 1 to 100 mouse embryonic stem cells, with less overestimation of 5mC level and higher genome coverage than conventional BS-seq. Additionally, UBS-seq quantitatively maps RNA 5-methylcytosine (m^5^C) from low inputs of mRNA and allows the detection of m^5^C stoichiometry in highly structured RNA sequences. Our UBS-seq results identify NSUN2 as the major ‘writer’ protein responsible for the deposition of ~90% of m^5^C sites in HeLa mRNA and reveal enriched m^5^C sites in 5′-regions of mammalian mRNA, which may have functional roles in mRNA translation regulation.

5-Methylcytosine (5mC) in DNA is a fundamental epigenetic mark having crucial roles in regulating gene expression, chromatin organization and cellular identity^1^. It is found in a wide variety of organisms and diverse cell types^2^, making it a valuable target for research in many areas of biology, including genetics, epigenetics and developmental biology. Bisulfite sequencing (BS-seq)^3^ is the gold standard for 5mC mapping because BS reagents are cost-effective and nonhazardous, and PCR amplification and sequencing after bisulfite treatment ensure high sensitivity without complicated procedures^3^.

BS-seq relies on the conversion of every single unmethylated cytosine to uracil, but only cytosines in single-stranded DNA are susceptible to chemical attack by bisulfite, therefore denaturation of the double-stranded DNA (dsDNA) is critical^4^ to avoid high background or false positives resulting from the incomplete conversion. On the other hand, complete bisulfite conversion necessitates harsh conditions such as a long reaction time and elevated temperature, causing severe DNA damage and overestimation of the 5mC level due to the biased fragmentation at C sites^5^. Currently, there are at least 12 commercial BS kits available for 5mC mapping in DNA, with Zymo’s EZ DNA Methylation-Gold Kit (referred as the conventional BS condition) being the most often used one in the literature. Current BS-seq procedures for 5mC detection in DNA suffer from several limitations, which are as follows: (1) a long reaction time at elevated temperature, the conventional BS condition requires 10 min at 98 °C plus 150 min at 64 °C, which is not ideal for fast detection or diagnosis applications; (2) severe DNA damage, the long reaction time in the conventional BS treatment leads to degradation of the majority of the treated DNA during the conversion process; (3) incomplete conversion of C-to-U, particularly at high GC DNA regions or highly structured DNA such as mitochondrial DNA (mtDNA); (4) prolonged bisulfite treatment damages DNA preferentially at unmethylated cytosine sites via depyrimidination, resulting in an overestimation of the methylation level; and (5) limited 4mC-to-U conversion ratio may generate false positives for genomes containing 4-methylcytosine (4mC). Despite the recent development of bisulfite-free 5mC sequencing methods such as enzymatic methyl-seq (EM-seq)^6^ and TET-assisted pyridine borane sequencing (TAPS)^7^ to address the issue of DNA degradation, they both include an additional enzymatic treatment step, which may lead to a decrease in conversion efficiency, increased operational complexity and variability between batches.

In addition to DNA, 5mC also exists in diverse RNA species including rRNA, tRNA, mRNA and various noncoding RNAs. Previous studies^8-11^ have revealed that RNA m^5^C modification and its effector proteins impact diverse cellular functions and have crucial roles in the etiology of bladder cancer^12^, hepatocellular carcinoma^13^, glioblastoma multiforme^14^ and leukemia^15^. However, the exact level and stoichiometry of m^5^C on different RNA species have been a subject of debate due to the absence of a sensitive, robust and quantitative sequencing method. Methods requiring antibody enrichment, such as RNA immunoprecipitation followed by deep sequencing (m^5^C-RIP-seq)^16^ and 5-azacytidine-mediated RNA immunoprecipitation^17^, cannot provide single-base resolution and m^5^C stoichiometry information, while cross-linking and immunoprecipitation (miCLIP)^11^ requires overexpressing the mutant enzyme. These approaches may not capture m^5^C sites embedded in highly structured RNA species. In recent years, BS-seq has been increasingly used to examine m^5^C modification^8,12,16,18^. Several commercial RNA BS conversion kits are available, including the EZ RNA Methylation Kit from Zymo Research and the Methylamp RNA BS Conversion Kit from Epigentek. Although BS-seq using these kits could be readily applied for m^5^C detection in abundant RNAs such as tRNA and rRNA^19,20^, the structure of some of these RNA species hampers accurate quantification. Discrepancies were observed when conventional BS-seq was applied to low abundant RNA species like mRNA, with some studies detecting over 8,000 m^5^C sites in mRNAs^21^ while other studies discovering only a few sites^22^. More recent studies have reported only a few hundred m^5^C sites in human and mouse transcriptomes using an improved BS-seq method and a more stringent computational approach ^9,12^. These inconsistent findings have raised the need to develop more sensitive and robust methods for identifying and quantifying real m^5^C sites in mRNA^9^.

To overcome the limitations of conventional BS-seq in mapping 5-methylcytosine in DNA and RNA, here we report a method of ultrafast BS-seq (UBS-seq) for both DNA and RNA. Using the optimized recipes composed of ammonium salts of bisulfite and sulfite and performing the reaction at 98 °C for ~10 min, UBS-seq affords a substantially lower background than the conventional BS condition, particularly in genomic regions with high GC content or highly structured such as mtDNA. UBS-seq also causes less DNA damage than the conventional BS condition due to the substantially shortened reaction time, and it mediates quantitative 4mC-to-U conversion to prevent false positives at 4mC sites. When applied to RNA m^5^C mapping, UBS-seq dramatically reduced the background to minimize false positives as compared with previous approaches. We identified thousands of m^5^C sites in HeLa mRNA, with ~90% of them being sensitive to NSUN2 depletion and a small fraction being NSUN6 substrates. By examining the distribution of the m^5^C sites in mRNA, we found that both HeLa and HEK293T mRNA exhibit the same characteristic of m^5^C site enrichment in 5′-UTR regions, suggesting that these m^5^C sites may regulate mRNA translation.

## Results

### Current BS-seq limitations and proposed solutions based on BS reaction mechanism

False positives and DNA/RNA damage are the two major limitations of the current BS-seq and have the following drawbacks: (1) severe DNA degradation results in fragments with smaller sizes. After C-to-U conversion the sequence complexity is further reduced, causing mapping challenges; (2) false positives can be generated by incomplete C-to-U conversion under the conventional BS condition, mainly due to incomplete denaturation of dsDNA or local secondary structure of RNA. To reduce DNA/RNA degradation, milder BS conditions are usually preferred, which may lead to suboptimal C-to-U conversion. These two opposing challenges have caused problems for 5mC mapping in DNA and hampered the accurate mapping of m^5^C in RNA. We attempted to overcome these challenges as follows: (1) shortening reaction time to reduce DNA/RNA degradation; and (2) raising reaction temperature to denature DNA/RNA to achieve complete C-to-U conversion.

**Fig. 1 ∣:**
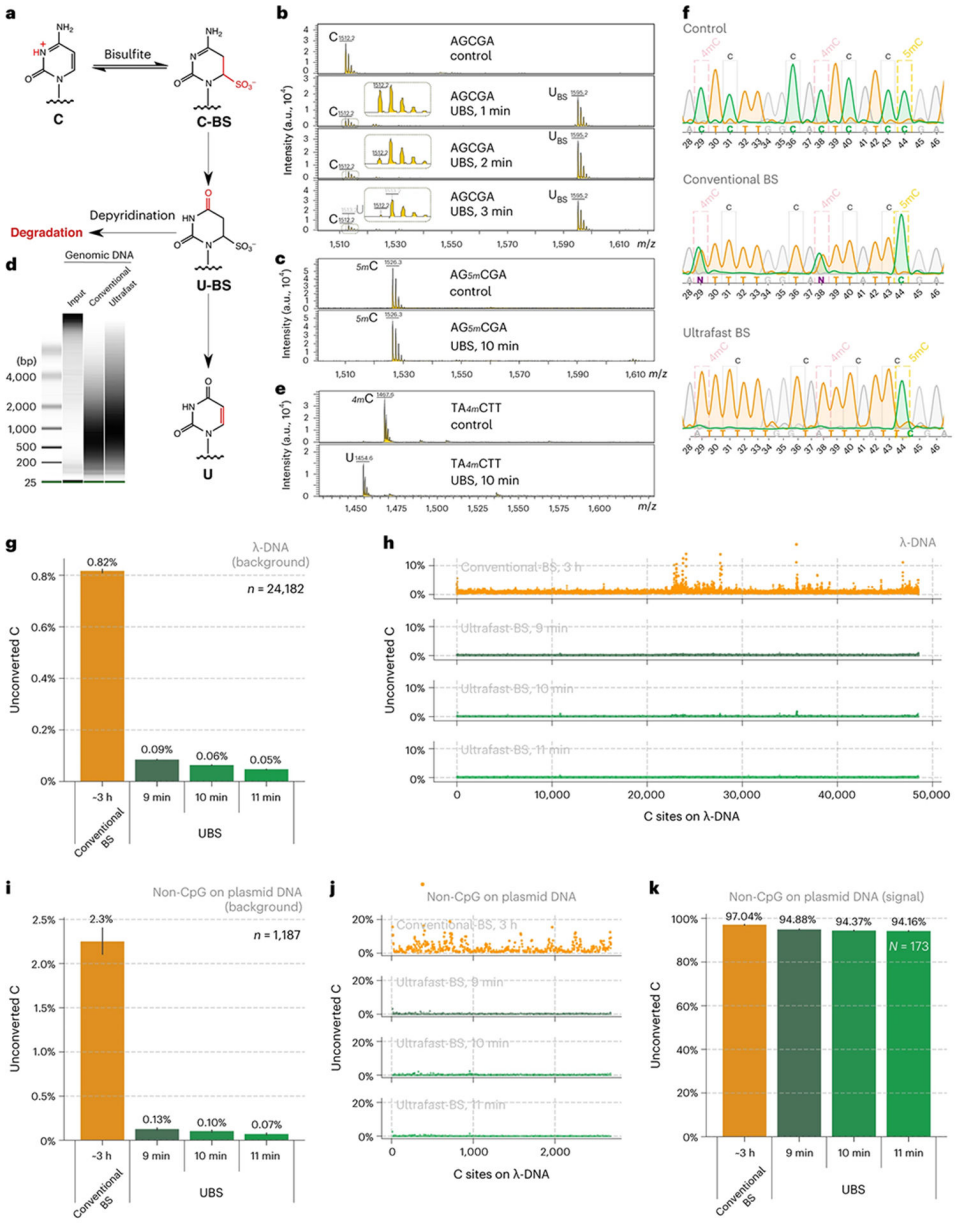
UBS-seq of DNA 5mC. **a**, Mechanism of BS-seq and BS-induced DNA degradation. **b**, The BS reaction of a DNA probe (5′-AGCGA) was monitored by MALDI-TOF MS. The peaks at 1,512, 1,595 and 1,513 were assigned to DNA probes containing unconverted C, U-BS and final product U, respectively. **c**, The DNA probe containing a 5mC modification (5′-AG5mCGA) showed no visible reaction with UBS-1 treatment. The peak at 1526 represented unreacted 5mC-containing probe, and no peak representing the corresponding product was observed at 1,609. **d**, DNA damage caused by UBS-seq treatment was much less than that caused by the conventional BS-seq condition. **e**, The UBS-seq condition achieved near quantitative 4mC deamination. The peaks at 1,467 and 1,455 were assigned to the DNA containing unconverted 4mC and the deaminated product U, respectively. **f**, Both C and 4mC were read as T, while 5mC was read as C based on Sanger sequencing results after UBS-1. As comparison, the 4mC bases were read as C and T in a 1:1 ratio under the conventional BS-seq condition. **g**, Optimization of the reaction time of UBS-seq by constructing and sequencing λ-DNA libraries after treatment for 9, 10 and 11 min, respectively. A sequencing coverage cutoff of 30 reads was applied, and all C sites identified across any of the four conditions were analyzed (*n* = 24,182). The average of the unconverted ratios is displayed above each bar, and the error bars represent the 95% confidence interval. **h**, The distribution of unconverted ratio along the λ-DNA genome after the conventional BS treatment and UBS-1 treatment for 9, 10 and 11 min, respectively. **i**, The converted ratio of all C sites on non-CpG motifs (*n* = 1,187) in plasmid DNA libraries following treatment with both conventional BS and for UBS at 9, 10 and 11 min, respectively. The average of the unconverted ratios is displayed above each bar, and the error bars represent the 95% confidence interval. **j**, The distribution of unconverted ratio of unmodified C sites (non-CpG) along plasmid DNA under various treatment conditions. **k**, The converted ratio of 5mC sites on CpG motifs (*n* = 173) in plasmid DNA after different BS treatments. The average of the unconverted ratios is displayed above each bar, and the error bars represent the 95% confidence interval.

Mechanistically, two competing pathways exist in BS-seq, with one giving the desired C-to-U conversion, whereas the other leading to the undesired DNA/RNA degradation (Fig. 1a)^23^. The protonated N3 nitrogen under acid conditions facilitates cytosine’s reaction with BS to give the C-BS adduct, which is converted to the U-BS adduct by deamination. Subsequent desulphonation of the U-BS adduct under basic conditions generates U, completing C-to-U conversion. Alternatively, the U-BS adduct may undergo spontaneous depyrimidination to cause DNA degradation^23^. Because BS reagent is involved in both steps of C-BS formation and subsequent deamination^24,25^, we speculated that the BS conversion rate should be accelerated if BS reagents with higher concentration were used^24^, which could allow the BS reaction to complete within a brief time and thus reduce DNA degradation. Furthermore, we reasoned that a higher temperature can not only accelerate BS reaction (Fig. 1a) but also assist in denaturing dsDNA or the secondary structures in RNA so that a complete bisulfite conversion could be accomplished within a much shorter period of time. Although higher BS concentration and higher reaction temperature might cause more DNA/RNA degradation, we reasoned that a much shorter reaction time could ultimately reduce degradation. In the meanwhile, we also need to ensure that increased concentration of BS and elevated reaction temperature do not result in undesired BS reaction with 5mC or m^5^C, which would reduce the sensitivity of 5mC/m^5^C detection.

### A UBS condition quantitively deaminates C within 3 min while keeping 5mC intact

Current BS treatments are usually conducted at ~3–5 M bisulfite concentration due to the limited solubility of sodium salts of bisulfite in water. Previously, it has been discussed in refs. 26,27 that ammonium bisulfite has much higher solubility in water, and in these studies, a mixture of ammonium bisulfite, sulfite and sodium bisulfite was used to obtain a ~10 M bisulfite reagent (2.08 g NaHSO_3_, 0.67 g ammonium sulfite monohydrate in 5.0 ml 50% ammonium bisulfite) to speed up DNA 5mC sequencing^26,27^. While attempting to reproduce the experiment, we observed that the mixture prepared according to this recipe required incubation at an elevated temperature to dissolve solids entirely, and the bisulfite salts readily precipitated out of the solution while it was cooling down. In addition, the solution was viscous and could hardly be transferred using a pipette, making it difficult to handle. We, therefore, decided to develop BS recipes consisting of ammonium salts of bisulfite and sulfite only^25^.

**Extended Data Fig. 1 ∣:**
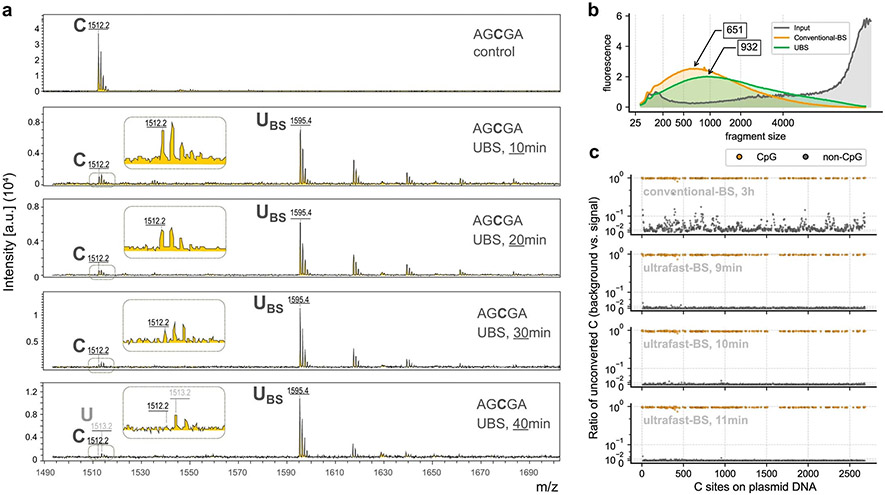
BS conversion efficiency of conventional BS-seq and UBS-seq on DNA. **a**) The progress of BS reaction of a DNA probe (5′-AGCGA) was monitored by MALDI-TOF MS, and the results showed that quantitative BS conversion required 40 minutes to complete (n = 1). The peaks at 1512.2, 1595.4, and 1513.2 were assigned to DNA probes containing unconverted C, U-BS and final product U, respectively. The inset figure provides a close-up view of the peaks near the unreacted oligo probe. **b**) The DNA damage assay revealed that gDNA treated with UBS condition exhibited significantly less damage compared to DNA treated with the conventional BS condition (Zymo EZ DNA Methylation Kits). **c**) The distribution of unconverted ratio of both CpG and non-CpG sites along plasmid DNA was examined under various treatment conditions.

We screened a series of BS conditions and identified a BS recipe (UBS-1), consisting of 10:1 (vol/vol) 70% and 50% ammonium bisulfite. We incubated a 5-mer DNA oligo AGCGA (Supplementary Table 1) with UBS-1 at 98 °C, and matrix-assisted laser desorption ionization time-of-flight mass spectrometry (MALDI-TOF MS) showed that within 3 min cytosine was completely converted to the uracil-BS adduct (Fig. 1b). In contrast, 40 min was required for complete conversion when the conventional BS treatment condition was used (Extended Data Fig. 1a), suggesting that our UBS condition can accelerate BS conversion ~13-fold. We next treated the corresponding 5mC DNA oligo probe under the same conditions and found that no visible reaction of 5mC was observed after 10 min treatment, suggesting that the UBS condition will not generate false negatives within 10 min (Fig. 1c).

### The UBS condition causes less DNA degradation

Severe DNA degradation is a hallmark of the conventional BS treatment. The long incubation time used in conventional BS-seq led to the degradation of over 90% of the incubated DNA^28^. Such extensive degradation could be problematic, especially for low-input DNA when the starting amount of DNA is often limited such as cell-free DNA (cfDNA). Given that the DNA degradation was mainly caused by the U-BS adduct, we reasoned that shortening the reaction time should reduce DNA degradation. We treated genomic DNA (gDNA) under the conventional BS condition and UBS-1 condition side by side. After desulphonation, a gel assay clearly showed that our UBS-1 condition induced less DNA degradation (Fig. 1d).

### The UBS condition quantitatively deaminates 4mC

4mC is another known cytosine methylation in bacterial gDNA, but it was also detected in eukaryote gDNA recently^29^. We previously showed that the conventional BS condition could only mediate ~50% 4mC deamination^30^; thus 4mC sites present in DNA might generate false positives in 5mC detection using conventional BS-seq in certain eukaryotic genomes. We asked whether the higher temperature and the higher BS concentration used in UBS-seq could facilitate the deamination of 4mC as well. We treated a short DNA oligo containing a 4mC modification (Supplementary Table 1) under UBS-1 condition, and MALDI-TOF MS showed that 4mC was quantitatively converted to dU (Fig. 1e). To further compare the deamination efficiency of C, 5mC and 4mC, we synthesized a DNA oligo containing C, 5mC and 4mC sites and applied our UBS condition side by side with the conventional BS condition. After PCR, Sanger sequencing results showed that both 4mC and C sites were all quantitatively read as T while the 5mC site remained as C after treated. In contrast, the two 4mC sites were read as C/T in a ~1:1 ratio under the conventional BS condition (Fig. 1f), indicating that the UBS condition can remove the potential false positives caused by 4mC.

### UBS-seq gives a much lower background than conventional BS-seq

To further evaluate false-positive rates in UBS-seq, we sonicated λ-DNA without 5mC modification and the pUC19 plasmid as a structured DNA control and a positive control for highly modified CpG motifs. After library construction using dsDNA ligation followed by UBS-1 or conventional BS treatment, respectively, the sequencing results showed that 10 min treatment under UBS-1 condition led to a background with the average unconverted rate of C as low as ~0.06%, while 11 min treatment only further reduced the background slightly. In contrast, the conventional BS treatment gave more than 13-fold higher background on λ-DNA (Fig. 1g). This high average unconverted rate was caused by clustered regions showing a much higher unconverted rate (~10%) under the conventional BS condition (Fig. 1h). In contrast, UBS-seq results showed evenly distributed C sites with low unconverted rates (Fig. 1h). For the more structured PUC19 plasmid DNA, the average unconverted rate for non-CpG sites increased to 2.3% in conventional BS-seq, more than 22-fold higher than that in UBS-1 condition (Fig. 1i,j). For all the methylated CpG sites, both methods detected >94% fraction on average (Fig. 1k). These results are consistent with our hypothesis that higher temperatures (98 °C) used in UBS-seq can effectively denature dsDNA in all regions to afford every low and evenly distributed background.

### Validation of UBS-seq using mESC gDNA

**Extended Data Fig. 2 ∣:**
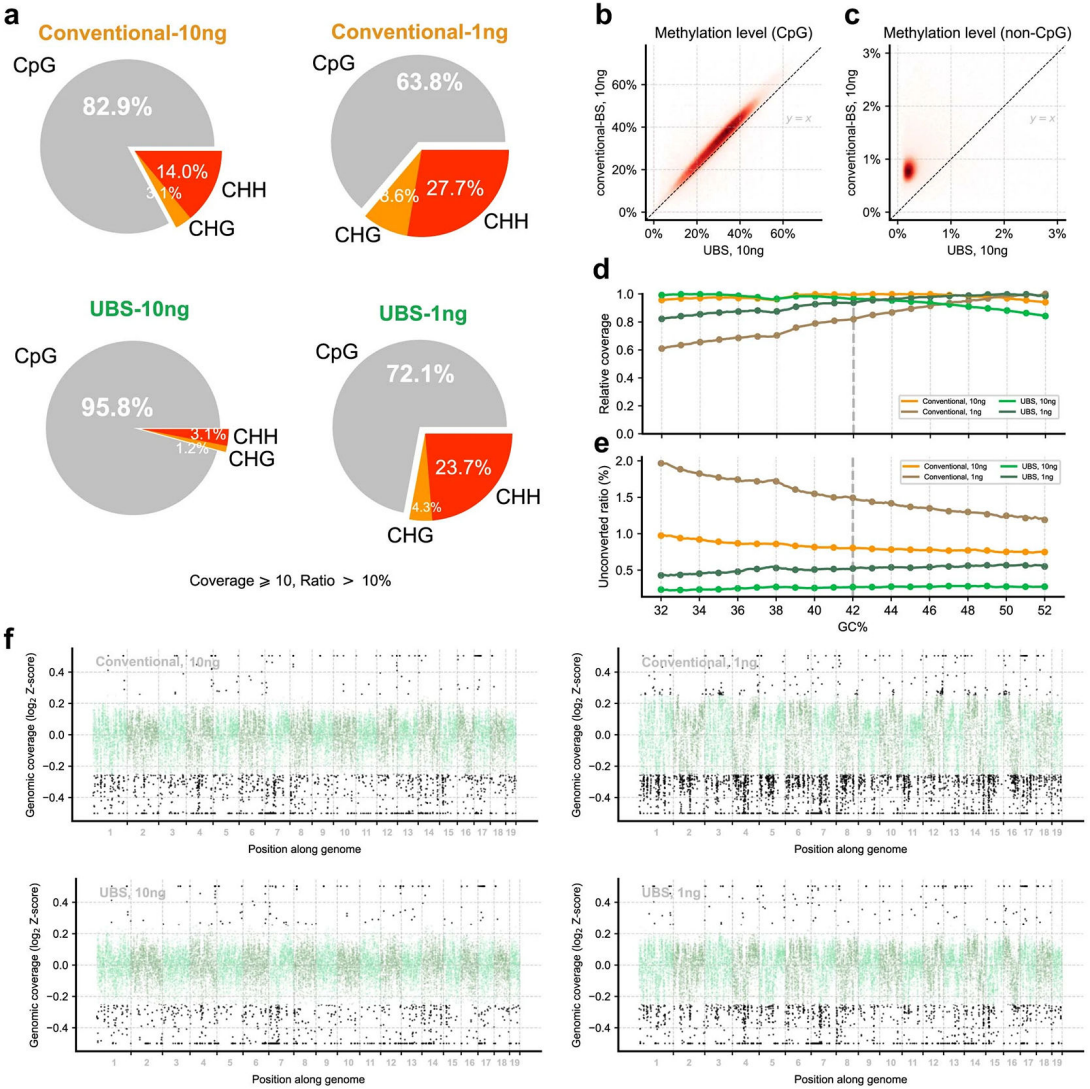
Comparison of UBS-seq and conventional BS-seq using mESC gDNA. **a**) With a cutoff ≥10× coverage and >10% of unconverted ratio, the number of the 5mC sites on non-CpG motifs detected by conventional BS-seq was twice as many as that detected by UBS-seq. **b**) The methylation levels detected in conventional BS-seq exhibited a slight increase at CpG sites in the 10 ng samples when compared to those in UBS-seq. **c**) Greater increases were observed in non-CpG sites. **d**) The sequencing coverage relative to a 1 kb sliding window was plotted against the GC% of the reference sequence in this region. The results indicate that conventional BS-seq exhibits greater coverage bias compared to UBS-seq. **e**) The unconverted ratio on non-CpG sites (CHG/GHH) show a slight decrease of unconverted ratio in regions with higher GC content for the both 10 ng and 1 ng UBS-seq libraries and the 10 ng conventional BS-seq library, while the 1 ng conventional BS-seq library shows an increase in the unconverted ratio in higher GC content regions. Comparison of the 10 ng and 1 ng samples of conventional BS-seq indicates that the conversion ratio at high GC regions of the lower input ratio is relatively higher. **f**) Comparison of genomic DNA coverage of DNA libraries prepared by conventional BS-seq and UBS-seq starting from 10 ng and 1 ng mESC gDNA, respectively. The average sequencing depth of sites within each 10 kb window was calculated and scaled to Z-score using the overall genomic coverage and plotted on a logarithmic scale. Outliers, indicated by black dots, were defined as values with an absolute Z-score greater than 1.5.

**Fig. 2 ∣:**
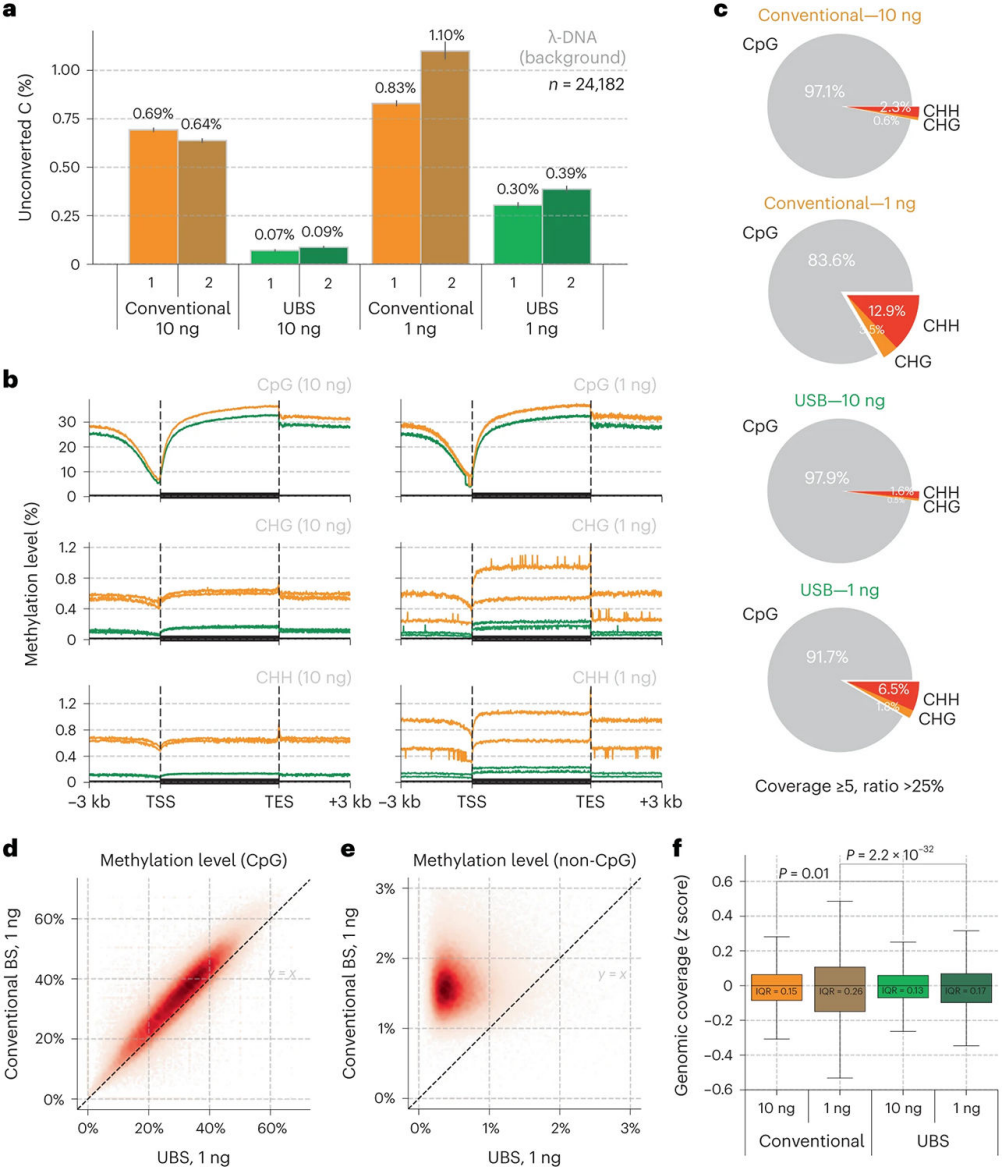
Comparison of UBS-seq and conventional BS-seq using low-input genomic DNA samples. **a**, Comparison of unconverted rate of unmodified C sites on spike-in λ-DNA (*n* = 24,182) between UBS-seq and conventional BS-seq in libraries starting from 10 and 1 ng mESC gDNA, respectively. The average of the unconverted ratios is displayed above each bar, and the error bars represent the 95% confidence interval. **b**, Comparison of the detected 5mC distribution pattern in mESC gDNA by both UBS-seq (green) and conventional BS-seq (orange) at CpG, CHG and CHH sites, respectively. **c**, When cutoff of ≥5 sequencing coverage and >25% unconverted ratio for 5mC sites detection was applied, conventional BS-seq showed that ~3% of all methylation sites occur to non-CpG sites on mESC gDNA library (10 ng) and the proportion increased to ~16% with a lower input amount (1 ng). In contrast, the UBS-seq treatment afforded smaller proportions of non-CpG sites, which were ~2% for 10 ng and ~8% for 1 ng samples, respectively. **d**, Correlation of the detected methylation level of all CpG sites among all the 10 kb genomic bins in 1 ng mESC gDNA libraries by both UBS-seq and conventional BS-seq at CpG sites. **e**, Correlation of the detected methylation level of all non-CpG sites among all the 10 kb genomic bins in 1 ng mESC gDNA libraries by both UBS-seq and conventional BS-seq. **f**, UBS-seq generated more evenly distributed coverage along the genome. The IQR was used to represent the statistical variance of the data. The box shows the IQR of the data, with a line at the median. The whiskers extend from the box to the 1.5× IQR of the data. When compared to conventional BS-seq, the UBS-seq method showed a 13% and 35% decrease in IQR value for 10 ng and 1 ng samples, respectively. Levene test for equality of variances yielded *P* values of 0.01 and 2.2 × 10^−32^ for 10 ng and 1 ng samples, respectively. IQR, interquartile range.

To comprehensively compare the performance of UBS-seq with conventional BS-seq, we constructed libraries starting from 10 and 1 ng mouse embryonic stem cell (mESC) gDNA, respectively. We first analyzed the background of all the C sites in spike-in λ-DNA and found that UBS-seq gave a much lower background than the conventional BS-seq (Fig. 2a). CpG sites represent the dominant motif for mammalian DNA methylation, while non-CpG methylation is thought to also have roles in gene regulation^31,32^. For CpG sites, both methods yielded similar 5mC distribution patterns, with conventional BS-seq showing slightly higher 5mC levels (Fig. 2b), but for the CHG and CHH sites, the detected 5mC levels by conventional BS-seq were notably higher than those by UBS-seq. Moreover, the variance between technical replicates became larger when applied to low-input samples such as 1 ng (Fig. 2b). Consequently, the percentages of the detected 5mC sites located in non-CpG motifs using conventional BS-seq were higher than those from UBS-seq (Fig. 2c and Extended Data Fig. 2a), suggesting that the detection of 5mC sites with low methylation stoichiometry using conventional BS-seq could lead to high false-positive rates.

Site-by-site comparison of methylation level within the 10 kb region also showed that conventional BS-seq gave systematical overestimation of the 5mC level (Fig. 2d,e and Extended Data Fig. 2b,c), suggesting that non-CpG motifs with lower modification levels are susceptible to biased degradation in conventional BS-seq, leading to an overestimation of non-CpG methylation levels in DNA. We calculated the average sequencing coverage and methylation level among all the 1 kb genomic bins and indeed observed a systematically reduced coverage and conversion at low GC content regions in the conventional BS-seq method while UBS-seq showed a more even distribution (Extended Data Fig. 2d,e). The genomic coverage analysis showed that UBS-seq gave more even genome coverage (Fig. 2f and Extended Data Fig. 2f). Together, our results demonstrated that UBS-seq outperforms conventional BS-seq in terms of reduced background, less 5mC level overestimation and higher genome coverage.

### Application of UBS-seq to low-input DNA samples and cfDNA

**Extended Data Fig. 3 ∣:**
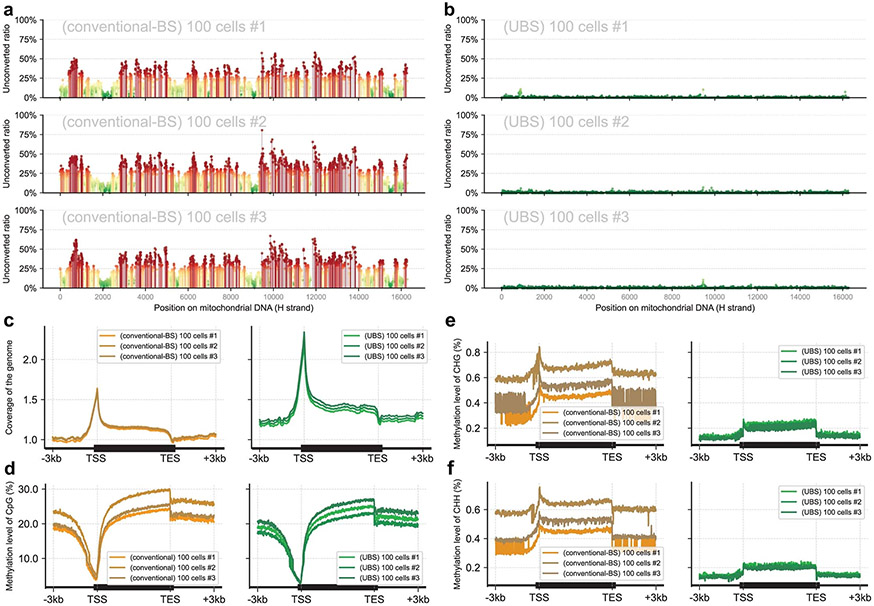
Comparison of UBS-seq and conventional BS-seq in application to lower input mESC samples. **a**) Unconverted rates of C sites along the heavy strand of mitochondrial DNA in three replicates of conventional BS-seq libraries are shown in different panels. Libraries were constructed starting from 100 mES cells. Higher ratios were colored in red, while lower ratios were colored in green. **b**) Similar to panel **a**, but the data for UBS-seq libraries were shown. **c**) Sequencing coverage across the gene body of samples starting from 100 cells, where both conventional BS-seq (left panel, n = 3) and UBS-seq (right panel, n = 3) exhibited similar distribution among three technical replicates. **d**) Comparison of the methylation levels on all CpG sites in three replicates of 100-cell experiments between the two methods. UBS-seq showed more consistent detection efficiency than conventional BS-seq. A signature of low methylation level near Transcription Start Sites (TSS) was observed in both conventional BS-seq and UBS-seq. **e**) In contrast to panel **d**, the detected methylation levels on CHG motifs were lower and the measured methylated level was more diverse among the three technical replicates of conventional BS-seq, while the measured methylation level was more consistent among UBS-seq samples. **f**) Similar to CHG motifs, detected methylation levels on CHH motifs were also more consistent among three technical replicates of UBS-seq.

**Fig. 3 ∣:**
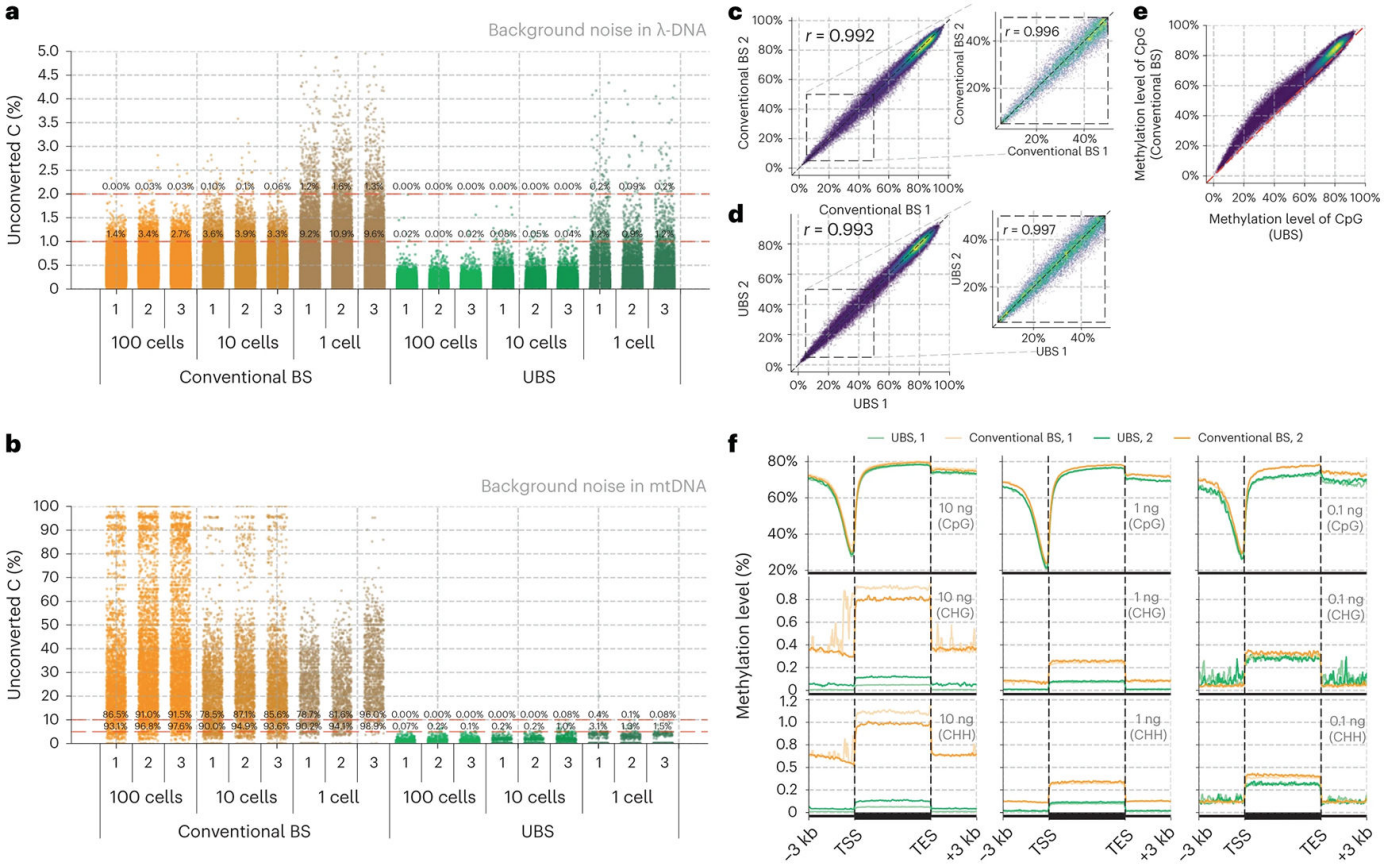
Application of UBS-seq to samples of 1–100 mES cells and cfDNA. **a**, Comparison of the background noise of the spiked-in λ-DNA without 5mC modification between UBS-seq and conventional BS-seq. The comparison was performed using input amounts of 100, 10 and 1 mES cell(s), respectively, and included three technical replicates (*n* = 3) for each condition. **b**, Comparison of the background noise of mtDNA (technical replicates *n* = 3) between UBS-seq and conventional BS-seq. **c**, Left, a high correction (*r* = 0.992) of the methylation level on CpG sites within a 10 kb slice window between two cfDNA replicates using conventional BS-seq. Right, the zoom-in of the partially methylated regions. **d**, The same analysis as **c**, but two replicates were treated with the UBS-1 reagent. **e**, The detected CpG methylation level in mESC gDNA libraries using conventional BS-seq was systematically higher than that using UBS-seq. Partially methylated sites showed bigger differences. For each sliding window, the methylation level was calculated as the average of two technical replicates (*n* = 2). **f**, The methylation levels of CpG, CHG and CHH sites within the gene body, along with 3 kb flanking regions before and after transcript end sites (TES). Two sets of samples, each with different input amounts of cfDNA (10, 1 and 0.1 ng), underwent treatment with either conventional BS (orange) or UBS (green).

Given that UBS-seq could generate a much lower background and cause less DNA degradation than conventional BS-seq, we used it to sequence 5mC starting from a small number of cells. We constructed libraries in triplicates starting from 100, 10 and 1 mES cell(s), respectively, using UBS-seq side by side with conventional BS-seq. As expected, background noise is a much bigger concern for 5mC mapping at single cell to 100 cells level when using conventional BS-seq. A dramatic increment in background noise was observed as the input sample amount decreased (Fig. 3a and Supplementary Table 2). Estimated from the spike-in λ-DNA, UBS-seq showed ~20-fold lower background than conventional BS-seq. Further analysis of mtDNA components in the libraries revealed that the background in mtDNA was much higher than that in spiked-in λ-DNA in all cases (Fig. 3b), probably due to the presence of highly structured regions in mtDNA. The proportion of unconverted sites reaches 90% even if the threshold of the unconverted rate of C was set to 10% in conventional BS-seq (Fig. 3b, Extended Data Fig. 3a,b and Supplementary Table 3). In contrast, UBS-seq showed much lower background, further demonstrating its advantage in reducing false positives. Furthermore, bisulfite treatment can only be conducted before DNA extraction when performing methylation profiling at the single-cell level, and the sequencing depth per cell is usually shallow. These factors may lead to an overestimation of heterogeneity between cells, especially for non-CpG regions (Extended Data Fig. 3c-e).

Recent development of 5mC detection in cfDNA as biomarkers showed great promise for cancer diagnosis, monitoring and prognosis^33,34^. Challenges limiting the application of BS-seq to cfDNA samples include the following: (1) for most human tissue, less than 0.5% of genomic blocks show >50% differential methylation^2^. A robust mapping method with good coverage is critical for detecting all differential sites; (2) cfDNA is a mixture of digested gDNA from different cell origins. Although the differential methylation pattern serves as an ideal signature for distinguishing diverse cell types, this difference comprises the admixture of DNA from multiple sources. To decouple the tissue methylation profiles and provide more accurate assignment, the detection method needs to have extremely low background and low quantification bias, vastly different from studying samples from a sole source; (3) usually small amount of cfDNA can be obtained from patients, making DNA degradation an even bigger challenge in conventional BS-seq. The advantages of UBS-seq over conventional BS-seq, such as much lower background (Fig. 2a), less DNA degradation (Fig. 1d), less bias and the ultrafast treatment time, indicate that UBS-seq can serve as a powerful tool for cfDNA 5mC sequencing.

**Extended Data Fig. 4 ∣:**
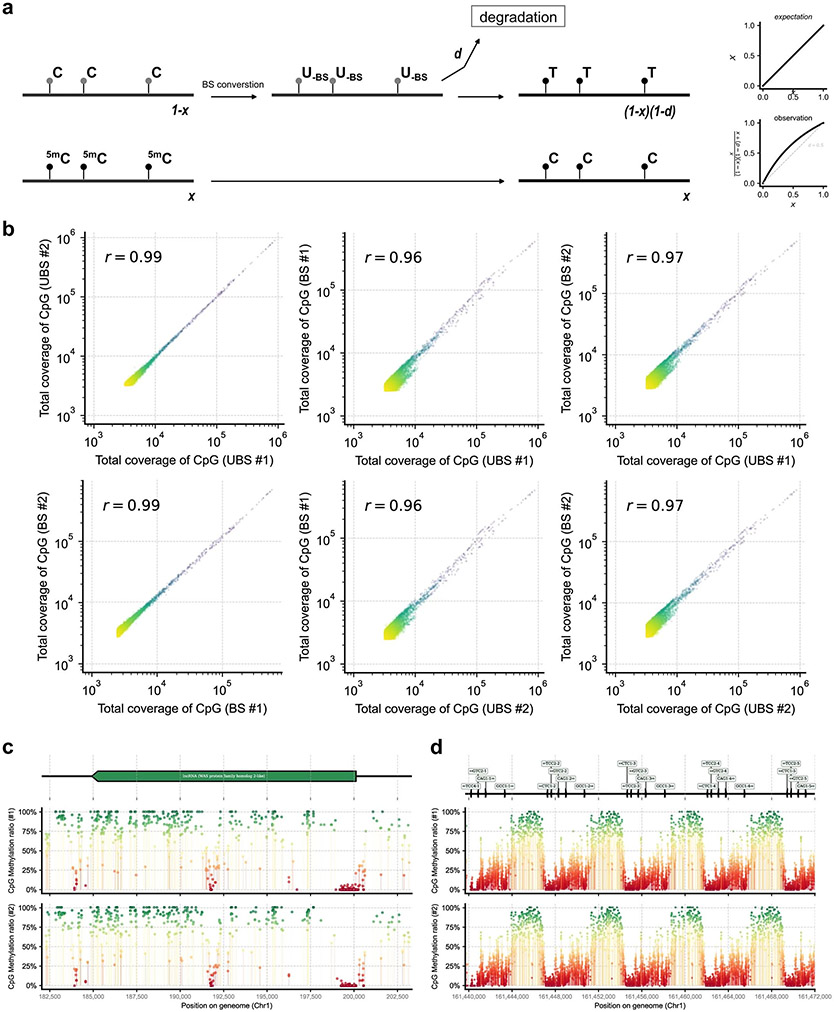
Comparison of UBS-seq and conventional BS-seq in application to cfDNA samples. **a**) For partially methylated regions, the proportion of methylated fragments was marked as x, while 1-x was the proportion of unmethylated fragments. BS treatments caused more degradation on reacted C sites and reduced the fraction of unmethylated fragments in sequencing libraries. The measurement of 5mC level would be affected by the degradation. Completely methylated and completely unmethylated sites showed less bias in the quantification results, while the stoichiometry of partially methylated sites could be over-estimated. **b**) Pairwise comparison of total sequencing coverage of all CpG sites within genomic windows (10 kb). Two replicates of conventional BS-seq samples (BS #1 vs. BS #2) and two replicates of UBS-seq samples (UBS #1 vs. UBS #2) both showed high correlation within methods (r = 0.99), but the correlation is slightly lower between methods (r = 0.96 ~ 0.97). **c**) Comparison of the methylated levels in two replicates of cfDNA UBS-seq samples within an example region. Highly methylated sites are colored in green and unmethylated/lowly methylated sites are colored in red. Detected 5mC ratios shown in y-axis were consistent between two replicates at single base resolution. **d**) Another example region showing consistency of methylation profile between two replicates of cfDNA UBS-seq libraries.

We constructed libraries using UBS-seq side by side with conventional BS-seq starting from 7.5 ng of purified human plasma cfDNA. Because the U-BS adduct causes most DNA degradation and unmethylated DNA fragments are more easily degraded than the methylated ones, the methylated fragments tend to be enriched, resulting in a potential overestimation of the overall 5mC level (Extended Data Fig. 4a). The total sequencing coverage of all CpG sites within genomic windows (10 kb) is highly consistent between two replicates of UBS-seq and those of conventional BS-seq, but a slight difference between the UBS and the conventional BS-treated samples was observed (Extended Data Fig. 4b), suggesting that the effects of DNA degradation are not uniform across genomic regions. Although the detected methylation level was quite consistent between replicates for both methods (Fig. 3c,d), a systematic higher detected methylation level in conventional BS-seq than UBS-seq was observed, and this difference was more pronounced in partially methylated regions (Fig. 3e and Supplementary Table 4). In addition, the methylation profiles across the gene body in two technical replicates of UBS-seq libraries are highly consistent (Extended Data Fig. 4c,d).

**Extended Data Fig. 5 ∣:**
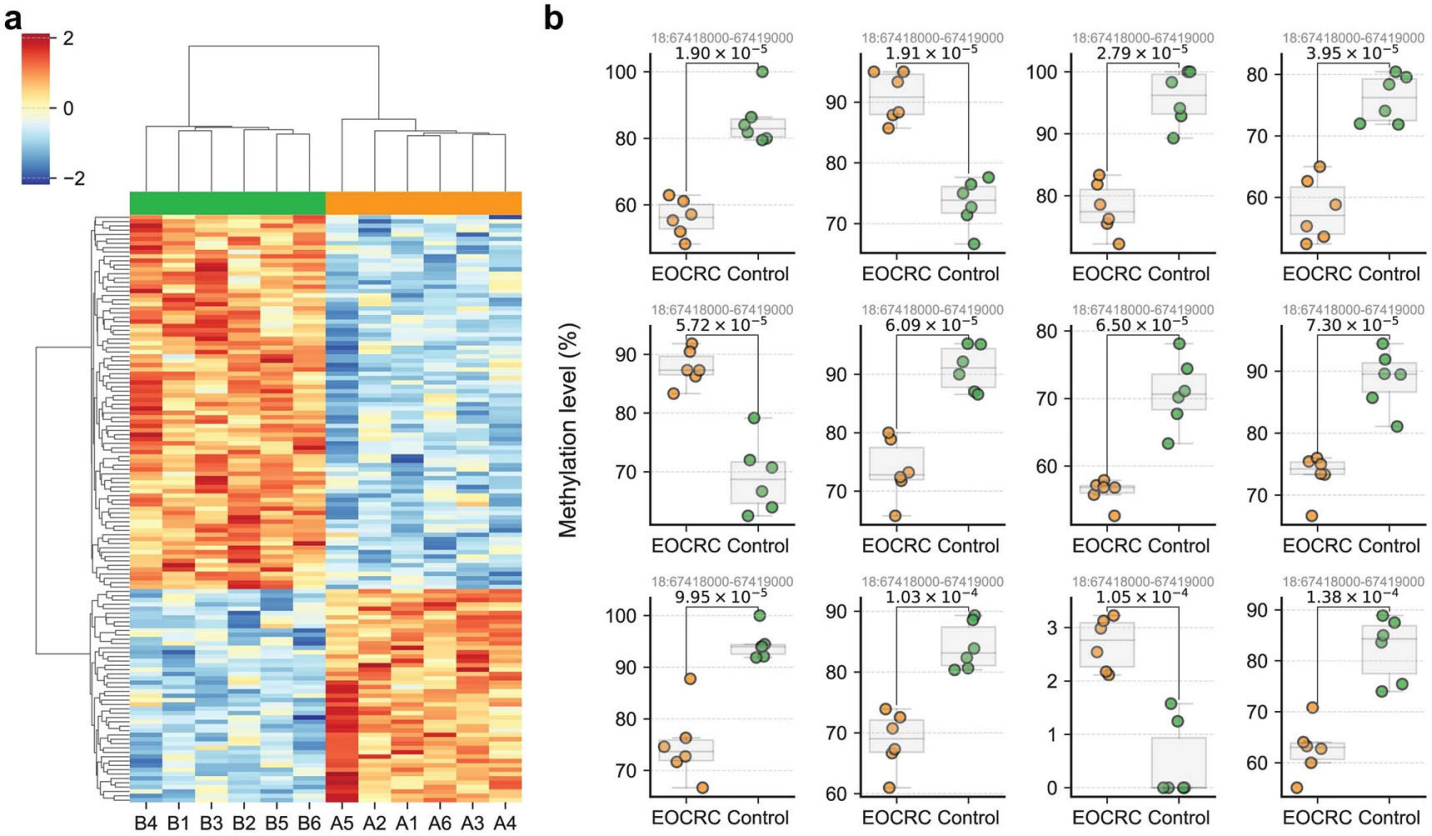
Potential application of UBS-seq to seek disease biomarkers for early-onset colorectal cancer (EOCRC) patients. **a**) Comparison of CpG site methylation levels within 1 kb windows between 6 EOCRC patients (A1 to A6) and 6 control samples (B1 to B6). UBS-seq libraries were prepared using 5 ng of cfDNA. Only CpG-rich regions with more than 20 CpG motifs were analyzed. Regions showing a significant increase or decrease (>20%) in methylation level with a p-value less than 0.001 were identified as putative biomarkers for EOCRC. The methylation levels of all differential regions (n = 135) were scaled by z-score and clustered based on their similarities. **b**) Comparison of the top 12 differential methylation regions. The genomic location of each region is labeled in the title of each panel. The p-values (T-test) shown on the figure indicate the significance of the difference between patients and healthy controls.

A hurdle in accurately determining cfDNA methylation arises from substantial variations in sample concentration and quality across different sources. These variations further undermine the robustness of library preparation. We further conducted parallel library construction using 10, 1 and 0.1 ng cfDNA isolated from pooled human plasma, respectively. UBS-seq consistently revealed a uniform distribution along gene body, both within technical duplicates and across different inputs (Fig. 3f). However, when using conventional BS-seq, detected 5mC stoichiometry at non-CpG sites exhibited greater variation, further supporting superior performance of UBS-seq when using cfDNA samples. We then applied UBS-seq to clinical samples from patients with early-onset colorectal cancer (EOCRC) and healthy controls to evaluate its potential as a diagnostic tool. In this proof-of-concept study, we successfully identified 135 putative differentially methylated regions as potential biomarkers (Extended Data Fig. 5a), allowing us to detect significant methylation level differences between patients and controls, even in regions where the differences in methylation levels are not particularly large (Extended Data Fig. 5b).

### Optimization of a UBS recipe for RNA m^5^C sequencing

**Fig. 4 ∣:**
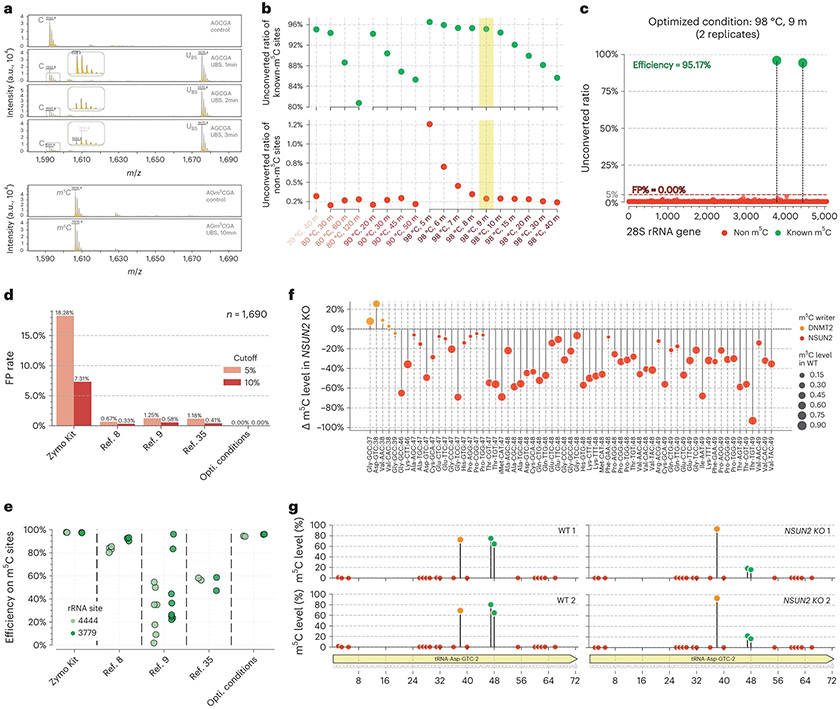
Optimization and validation of ultrafast bisulfite conditions using human 28S rRNA and tRNA. **a**, BS reaction of an RNA probe (5′-AGCGA) was monitored by MALDI-TOF MS. The peaks at 1,592, 1,675 and 1,593 were assigned to RNA oligo probes containing unconverted C, U-BS and final product U, respectively. As comparison, the RNA probe containing a m^5^C modification (5′-AGm^5^CGA) showed no visible reaction after 10 min of UBS-2 treatment. The peak at 1,606 represented unreacted m^5^C-containing oligo probe, and no peak representing the corresponding product was observed at 1689. **b**, Optimization of reaction time and temperature using 28S rRNA. The *y* axis is the average number of unconverted ratios for each condition based on two technical replicates (*n* = 2). The trade-off of the positive signal and the background noise indicated that 98 °C for 9 min is the best condition. **c**, The unconverted rate of all C and m^5^C sites along 28S rRNA. The detected m^5^C fractions at the two known m^5^C sites were over 95%, while no false positive (FP) was detected using the UBS-seq protocol when the detection threshold was set to 5% (FP% = 0). The original numbers for each site in two replicates (*n* = 2) are presented together. **d**, Comparison of the FP rate at non-m^5^C-modified C sites on ribosomal RNA (*n* = 1,690) among Zymo EZ RNA Methylation Kit, the three previously reported protocols^8,9,35^ and the UBS-seq condition (*n* = 2). **e**, Comparison of the detected fractions of the two m^5^C sites on ribosomal RNA using the same data in the panel (**d**). While the detected fractions at the m^5^C sites dropped dramatically in the previously reported protocols with prolonged treatment, UBS-seq detected high stoichiometry at these m^5^C sites. **f**, tRNA m^5^C sites detected in WT and *NSUN2* KO cell lines (*n* = 2). The m^5^C sites deposited by DNMT2 were in orange and sites installed by NSUN2 were in red. The size of the dot represented the modification level in WT cells, and the *y* axis showed the change of m^5^C level upon *NSUN2* KO. **g**, Converted ratio of C sites along tRNA Asp^GTC^. The two replicates of the WT cells are on the left side, while the two replicates for *NSUN2* KO cells are on the right side. C sites, DNMT2-targeted m^5^C sites and NSUN2-targeted m^5^C sites were colored in red, orange and green, respectively. FP, false positive.

A major obstacle for RNA m^5^C BS-seq is to balance reaction temperature versus RNA degradation. Unlike mammalian gDNA, the mRNA m^5^C modification is far less frequent and the modification fraction level is also much lower^34^, therefore the high false-positive rate resulted from lower reaction temperature used for the convention BS-seq has been a major challenge in RNA BS-seq for detecting real m^5^C sites in mRNA^8,9,35^. Encouraged by the successful applications of UBS-seq to 5mC mapping in low-input gDNA and cfDNA samples, we reasoned that UBS condition should induce efficient C-to-U conversion in RNA within a short period of reaction time without causing severe RNA degradation. To test this, we synthesized the corresponding RNA oligo probe (5′-AGCGA) as a model to further optimize the UBS recipe. We found a slightly different BS recipe (UBS-2) that could mediate a quantitative conversion of C to U-BS adduct within 3 min at 98 °C (Fig. 4a). Under these conditions, we did not observe an obvious bisulfite reaction of m^5^C within 10 min of treatment (Fig. 4a), suggesting that the UBS-2 condition is suitable for RNA m^5^C detection.

**Extended Data Fig. 6 ∣:**
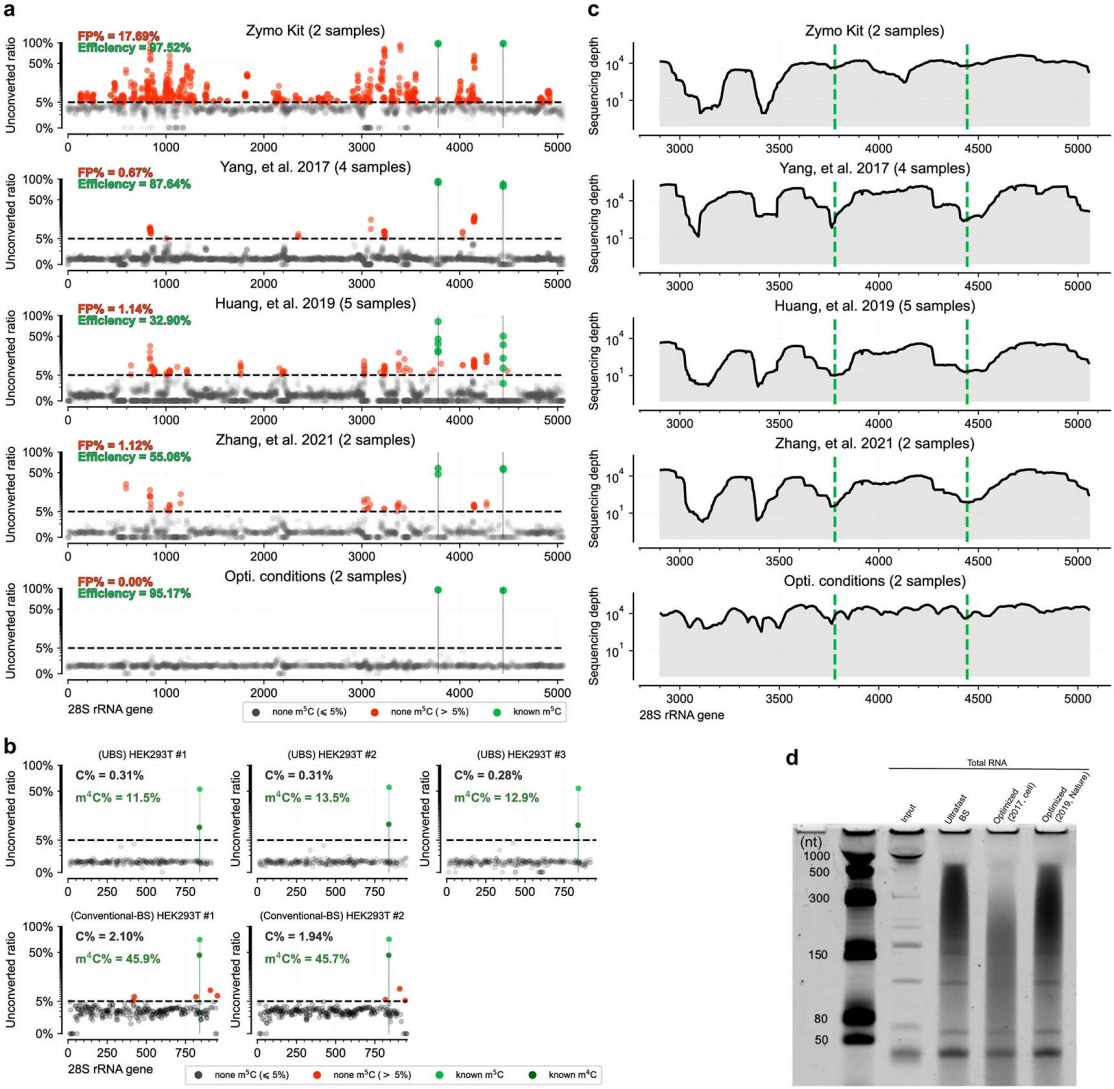
Comparison of UBS-seq with conventional BS-seq and three reported methods in RNA m^5^C sequencing. **a**) m^5^C sequencing data from libraries constructed with EZ RNA Methylation Kit (Zymo Research) and the three published studies^9,35,8^ were analyzed using the same pipeline and compared to those from UBS-seq protocol. The default condition of RNA bisulfite kit showed serious cluster effect on highly structured regions, and filtering m^5^C sites with a 5% cutoff showed 17.69% of false positive rate. The average detected fraction on the two known m^5^C sites was 97.52%. Similarly, false positive rate vs. efficiency in three previous studies^9,35,8^ were 0.67% vs. 87.64%, 1.14% vs. 32.90% and 1.12% vs. 55.06%, respectively. In contrast, no false positive was detected on 28 S rRNA using UBS-seq method, and the average detected fraction on the two known m^5^C sites was 95.17%. Two replicates were performed using the commercial kit and the UBS-seq condition (n = 2). **b**) A comparison was conducted on the m^4^C and m^5^C signals within the mitochondrial 12 S rRNA of HEK293T samples. The unconverted ratio at unmodified C sites was ~7 times lower in UBS-seq, while the rate of unconverted m^4^C sites was ~4 times lower in UBS-seq compared to the conventional BS-seq method. **c**) Comparison of the sequencing depth across 28 S rRNA using the same data in panel **a**. Conventional BS-seq showed low coverage at highly structured regions. Methods in the three published studies also showed decrease of sequencing coverage near m^5^C sites, resulting in lower sensitivity for m^5^C detection. **d**) Comparison of RNA degradation caused by different BS treatment.

We then targeted human 28S rRNA to further optimize reaction conditions because it contains two confirmed m^5^C sites. Our sequencing data showed that, as the background decreased with higher temperature and longer reaction time, the detected m^5^C fractions at the two known m^5^C sites also decreased (Fig. 4b). The optimal condition was achieved with incubation at 98 °C for 9 min, under which the average fraction for the two m^5^C sites detected in duplicates was >95%. Notably, no false-positive site was detected with a 5% cutoff for the unconverted rate of C (Fig. 4c and Extended Data Fig. 6a). In contrast, when EZ Zymo RNA Methylation Kit was used, a very high background was observed, resulting in a ~18% of false-positive rate (Fig. 4d) despite that the two known m^5^C sites could be detected with high fractions (Fig. 4e). In addition, we found that the m^4^C site in the 12S subunit mitochondrial rRNA showed higher converted ratio than that in conventional BS-seq (Extended Data Fig. 6b).

To make a comparison with other reported BS-seq conditions, we reanalyzed the raw sequencing data from literature using the same pipeline (Extended Data Fig. 6a). In a study discussed in ref. 8, a harsher BS condition was used to reduce the background; however, multiple false-positive C sites with ≥5% unconverted rates persisted in 28S rRNA (Fig. 4d). The long reaction time also led to decreased fractions detected for the two known m^5^C sites^8^ (Fig. 4e). In a study discussed in ref. 9, the Zymo RNA BS reagent was used and three rounds of BS treatment were conducted to further reduce background, but the detected m^5^C fractions also decreased dramatically^35^ (Fig. 4d,e and Extended Data Fig. 6a). To examine RNA damage caused by BS treatment, we treated the HeLa total RNA under the UBS-2 conditions side by side with literature BS conditions. After desulphonation, a page gel assay showed that UBS-seq caused less RNA damage (Extended Data Fig. 6c). Taken together, the UBS condition outperformed all the reported BS conditions in terms of lower background and higher sensitivity in detecting m^5^C sites in rRNA.

We next analyzed reads coverage by comparing reads distribution in UBS-seq with other methods^8,9,35^ (Extended Data Fig. 6d). The C-rich regions showed less sequencing depth in all published data, suggesting frequent RNA fragmentation at C sites during BS treatment. However, the fluctuation of the read depth is much less in UBS-seq compared with those from other methods, demonstrating that our UBS condition generated less RNA degradation and thus much less bias in estimating the m^5^C fraction at the modified sites. While a large amount of input RNA is usually required for longer reaction time and repeated treatments in previously reported procedures, our UBS-seq could dramatically reduce input RNA amount required.

### Detecting m^5^C sites in highly structured human tRNA by UBS-seq

Human tRNAs are highly structured. To avoid severe RNA degradation, other BS-seq methods treated RNA at 54 °C or 75 °C, which is insufficient to denature the secondary structures at highly structured regions of tRNA, causing high background and false positives. The m^5^C is present at sites 48, 49 or 50 in certain human tRNAs with NSUN2 as the methyltransferase. To detect m^5^C in tRNA, we constructed libraries using the small RNA fraction (<200 bp) of total RNA from the wild-type (WT) and *NSUN2* knockout (KO) A549 cell line. We expected that the m^5^C fraction at sites 48, 49 and 50 would be sensitive to NSUN2 depletion. The m^5^C site at C38 was selected as a control because it is installed by DNMT2, and thus its fraction should be insensitive to NSUN2 depletion. Indeed, sequencing results showed that the m^5^C fractions at sites 48, 49 or 50 all decreased dramatically upon NSUN2 depletion, while the detected m^5^C fraction at C38 remained unaltered (Fig. 4f and Supplementary Table 5). In addition, all the C sites in tRNA Asp^GTC^ displayed extremely low background, while the three m^5^C sites at 38, 47 and 48 showed modification fractions of ~80% in the WT samples (Fig. 4g). Upon NSUN2 depletion, the fraction dropped by >50% at sites 47 and 48. The more accurate and quantitative detection of these m^5^C sites in highly structured RNA by UBS-seq should facilitate future studies on the biological functions and dynamic tuning of m^5^C on tRNA and other RNAs.

### Application of UBS-seq to human mRNA

**Extended Data Fig. 7 ∣:**
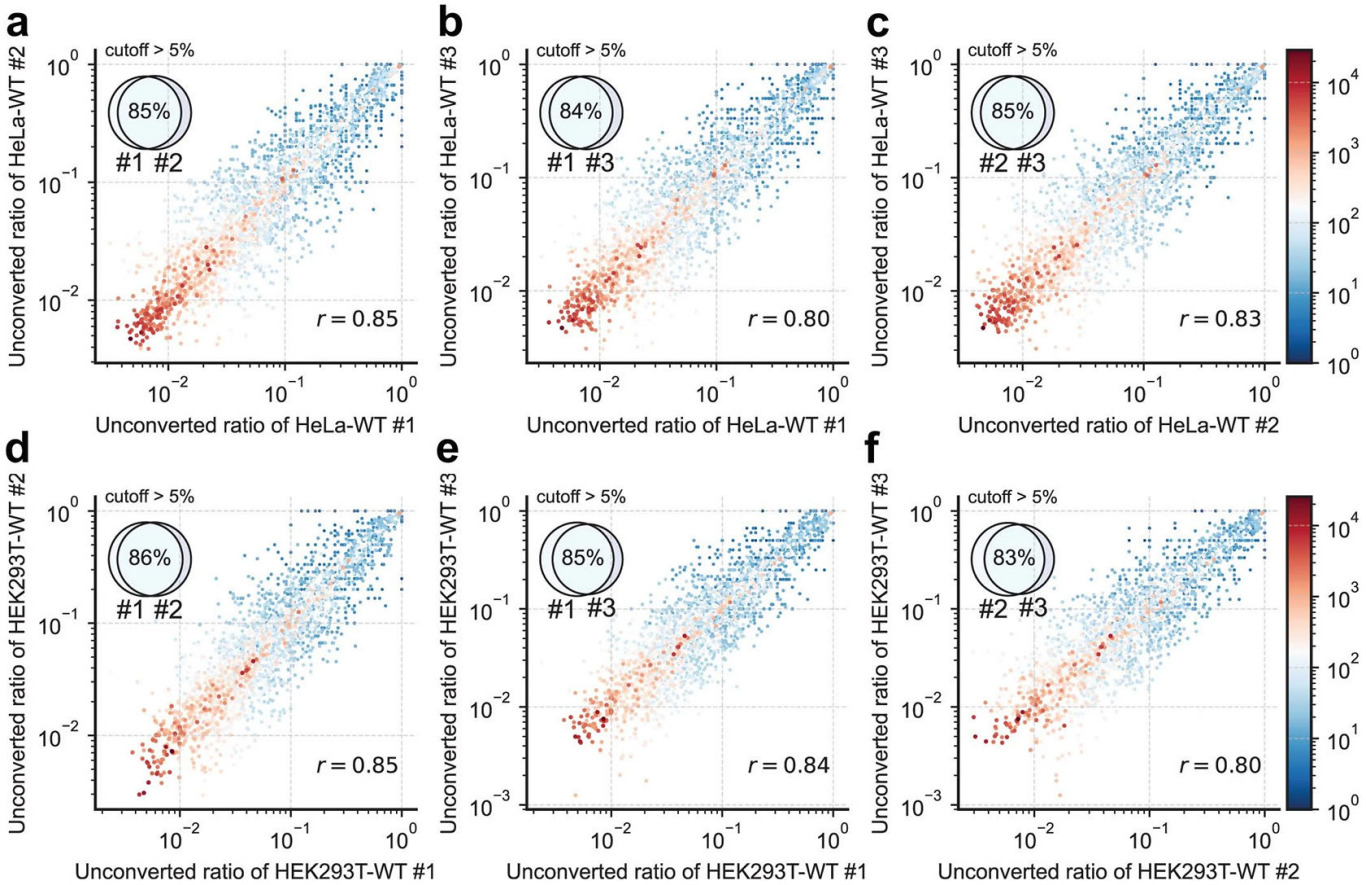
Reproducibility of RNA m^5^C UBS-seq libraries. **a**) The unconverted ratio of putative m^5^C sites in replicate 1 and replicate 2 of HeLa mRNA libraries is shown on the x and y axes, respectively. The Pearson’s correlation (*r* = 0.85) of the unconverted ratio in the two replicates was labeled. The minimal coverage in these two libraries is represented by different colors, with high coverage sites in red and low coverage sites in blue. The inset Venn plot illustrates the overlap ratio of two libraries in sites with an unconverted ratio greater than 5%. **b**) Similar to panel **a**, the overlap of replicate 1 and replicate 3 is shown. **c**) Similar to panel **a**, the overlap of replicate 2 and replicate 3 is shown. **d–f**) Similar to panel **a–c**, but the data for HEK293T mRNA sites was shown.

**Extended Data Fig. 8 ∣:**
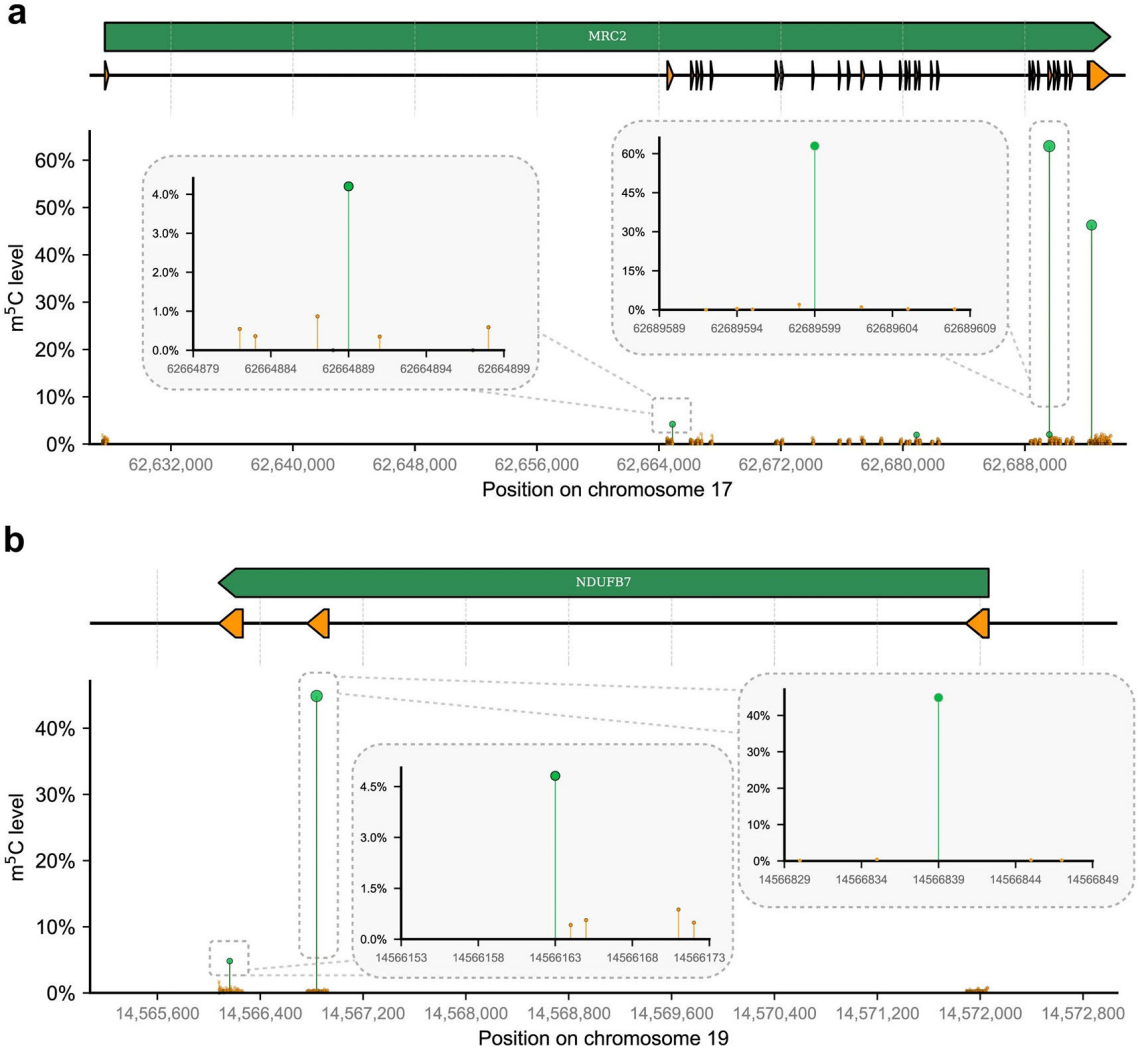
Signal-to-noise of detected m5C sites on mRNA. **a**) The scatter plot illustrates the unconverted ratio of detected m^5^C sites (in green) and all background C sites (in orange) on the MRC2 gene. The y-axis represents these ratios, while the x-axis displays the position of the site along the reference genome. Only sites with coverage exceeding 50 are displayed. The exon architecture of the coding gene is depicted in the diagram above the panel. Additionally, the inset plots highlight one m^5^C site with high-frequency and one with low-frequency, with the unconverted ratio of all C sites within a 20 nt flanking region indicated. **b**) Another example is presented to demonstrate the signal-to-noise ratio of the NDUFB7 gene.

**Fig. 5 ∣:**
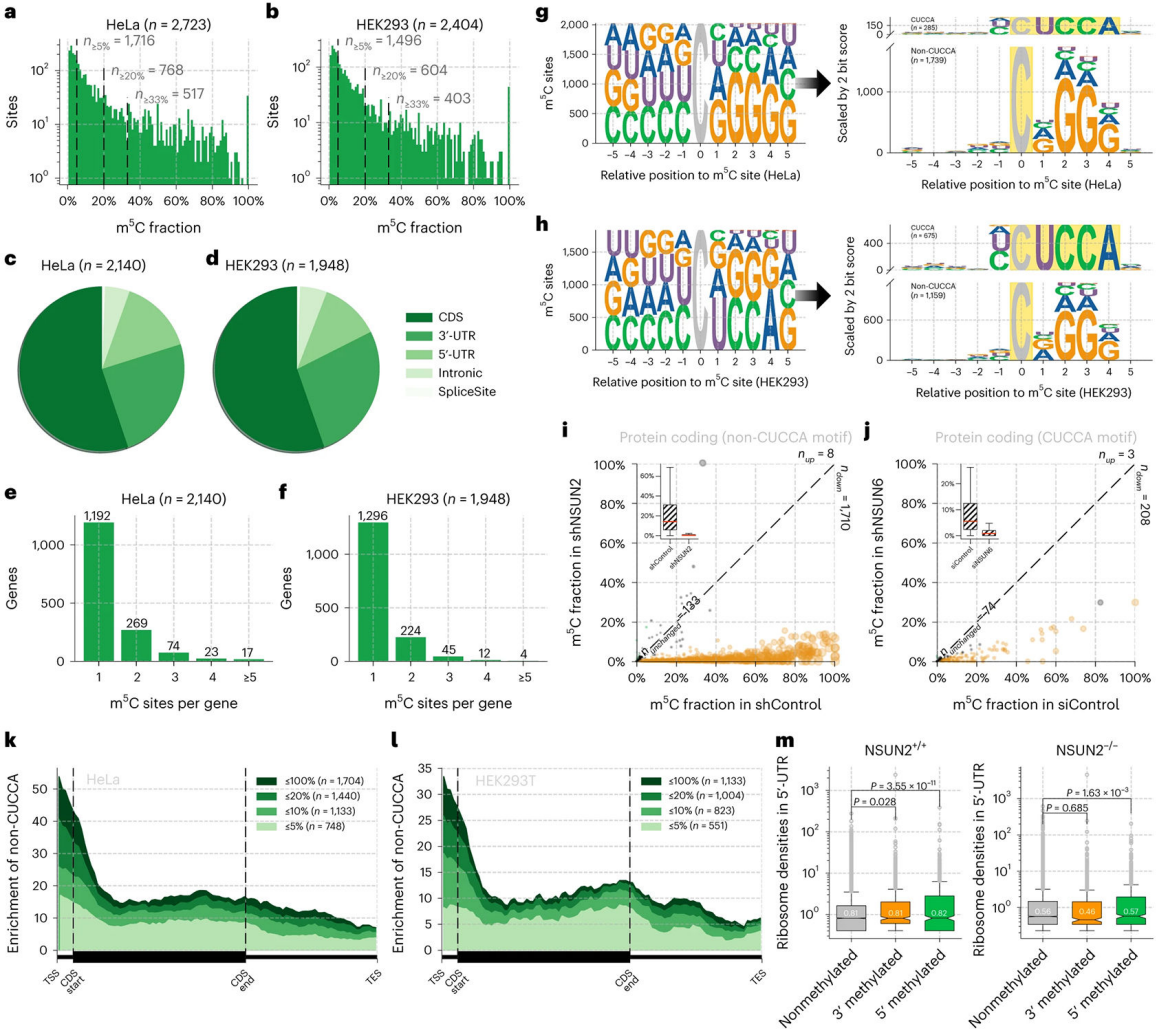
m^5^C sites detected by UBS-seq in mRNA from HeLa and HEK293T cells. **a**, Detected m^5^C site distribution with different modification fractions in HeLa mRNA. Of 2,723 detected sites (binomial test, *P* < 10^−6^), 1,716, 768 and 517 of them showed modification fractions ≥5%, 20% and 33%, respectively (*n* = 3). **b**, Distribution of m^5^C modification fraction in HEK293T mRNA. Of 2,404 detected sites (binomial test, *P* < 10^−6^), 1,496, 604 and 403 sites of them showed modification fractions ≥5%, 20% and 33%, respectively (*n* = 3). **c**, Distribution of m^5^C sites in relation to regions of coding genes in HeLa cells. **d**, Distribution of m^5^C sites in relation to regions of coding genes in HEK293T cells. **e**, Distribution of m^5^C sites per gene in HeLa cells. **f**, Distribution of m^5^C sites per gene in HEK293T cells. **g**, Motif enrichment of the detected m^5^C sites in HeLa mRNA. Sites were split into CUCCA and non-CUCCA groups based on the motif, and the height of the sequence logo was scaled by the maximum information content (2 bit) of the variable positions within the window, with *y* axis representing the number of detected m^5^C sites. **h**, Similar to **g**, motif enrichment for the detected m^5^C sites in HEK293T mRNA. **i**, The m^5^C fraction of non-CUCCA motif on protein-coding transcripts upon NSUN2 knockdown versus control. Orange dots represent sites showing more than two-thirds decrease of the measured m^5^C modification level. Green dot represents site showing more than threefold increase of the modification level. Gray dots represent sites without noticeable changes. In total, 1,710 sites show a significant reduction on methylation level, while only 133 sites remain unchanged (*n* = 2). The inset plot illustrates a comparison of the m^5^C fraction between two samples, using a box plot. The red line indicates the median and the whiskers extend from the box to 1.5× IQR of the data. **j**, Similar to i, the CUCCA motif upon *NSUN6* knockdown was shown. In total, 208 sites showed more than two-thirds reduction of the measured methylation level (*n* = 2). **k**, Distribution of m^5^C sites at the 5′ and 3′ ends of transcripts in HeLa cells. **l**, Distribution of m^5^C sites at the 5′ and 3′ ends of transcripts in HEK293T cells. **m**, In connection with ribosome profiling data, genes with m^5^C sites installed on the 5′ end (within the first one-third of the transcript) show significantly higher 5′-UTR ribosome binding densities than genes without m^5^C methylation (*P* = 3.55 × 10^−11^) in one-tailed *t* test. In contrast, genes with m^5^C sites located on the last two-thirds of the transcript did not show ribosome binding enrichment signal as high as 5′- end methylated ones (*P* = 0.028). Upon *NSUN2* mutation, the enrichment signal of 5′-UTR ribosome densities decreased with reduced statistical significance (*P* = 1.63 × 10^−3^). The median ribosome binding densities for each group are indicated on the box.

After optimizing and validating UBS-seq using human 28S rRNA and tRNA, we next constructed libraries using polyA^+^ RNA from HeLa and HEK293T cell lines in duplicates. Unlike mammalian gDNA, the mRNA m^5^C modification is far less frequent (the median modification level typically less than 10%^35^), making detection of these sites much more challenging. Additionally, the transcript levels of different genes can vary dramatically, which can cause bias in detecting low-expression genes with severe RNA degradation. To address these challenges, we developed statistical methods (Supplementary Note) to detect m^5^C sites on low abundant transcripts. Consistent with the existing strategies, reads with more than three unconverted sites, or the number of unconverted sites accounting for more than half of the converted sites, were eliminated first. The probability (*P*) of random errors at different sequencing depths was then calculated based on the binomial distribution. Only those sites with *P* value less than 10^−6^ were retained. This effectively avoids the preference for low-expression sites of existing detection algorithms because a random error causes more perturbation for low-coverage sites. After we applied these detection criteria together with a ≥5% unconverted cutoff, 2,723 and 2,404 m^5^C sites were identified in polyA^+^ RNA from HeLa and HEK293T cells, respectively (Fig. 5a,b and Supplementary Tables 6 and 7), and these m^5^C sites showed over 80% overlap between replicates (Extended Data Fig. 7a-f). We were able to detect many more m^5^C sites than the previous reports^9,35^ by applying UBS-seq and the statistic method, with good overlap with those reported (Supplementary Note). Both highly and lowly modified m^5^C sites exhibit a high signal-to-noise ratio along the protein-coding gene without significant cluster effect observed in flanking regions (Extended Data Fig. 8a,b), suggesting that the m^5^C sites detected by UBS-seq are high confidence ones. False positives caused by structured motifs, an issue commonly encountered in conventional RNA BS-seq methods, could be effectively suppressed.

**Extended Data Fig. 9 ∣:**
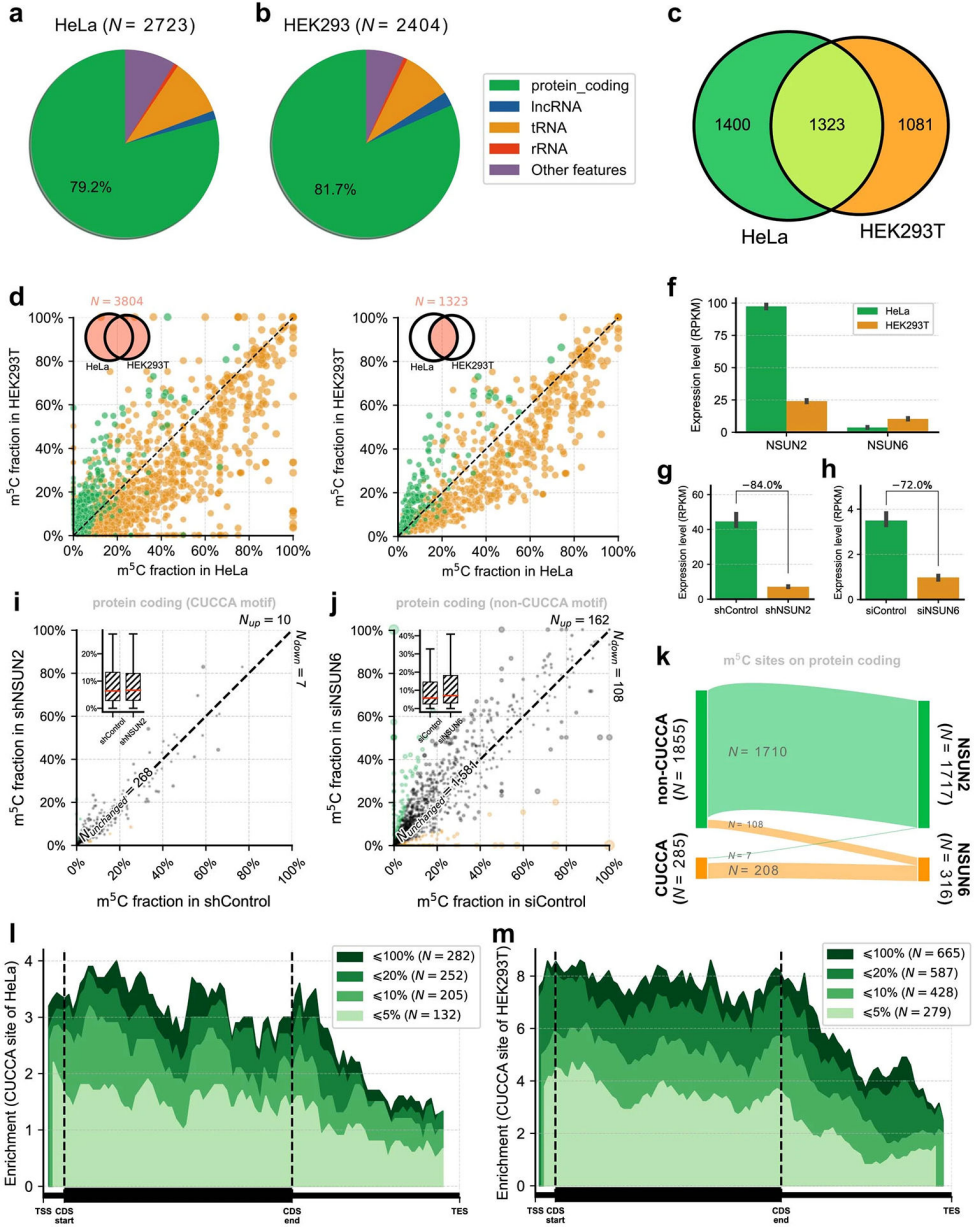
Detected m^5^C distribution in mRNA of HeLa and HEK293T cells. **a**) Distribution of m^5^C sites on different gene types in HeLa mRNA. Most of the detected sites (79.2%) were on protein coding genes. Sites from 3 technical replicates (n = 3) were used in this analysis. **b**) Similar to panel **a**, but distribution of HEK293T cell (n = 3) was shown. **c**) Overlap of the detected m^5^C sites in HeLa and HEK293T mRNA. **d**) Comparison of the modification fractions of all m^5^C sites detected in HeLa and HEK293T cells. Green dots denote sites on the CUCCA motif, while yellow dots denote sites on non-CUCCA motifs. CUCCA motifs showed higher modification fraction in HEK293T cells than in HeLa cells, while non-CUCCA motifs showed higher modification fraction in HeLa cells than in HEK293T cells. **e**) Similar to panel **d**, but the intersect of m^5^C sites were shown. **f**) The gene expression level of *NSUN2* and *NSUN6* in two cell lines was calculated based on the sequencing data. The expression level of *NSUN2* is higher in HeLa cell than in HEK293T cells, while *NSUN6* level is higher in HEK293T cells than in HeLa cells. **g**) Expression level of *NSUN2* in shNSUN2 cell line vs. shControl indicated that the knockdown efficiency of *NSUN2* was ~84%. **h**) Expression level of *NSUN6* in siNSUN6 cell line vs. siControl indicated that the knockdown efficiency of *NSUN6* was ~72%. **i**) In contract to Fig. 5i, m^5^C sites on CUCCA motif did not respond to *NSUN2* knockdown (n = 2 technical replicates). **j**) In contract to Fig. 5j, m^5^C sites on non-CUCCA motif did not respond to *NSUN6* knockdown (n = 2 technical replicates). **k**) A majority of m^5^C sites on protein coding genes could be assigned to either *NSUN2* or *NSUN6* gene. **l**) In contract to Fig. 5k, the detected m^5^C sites within the CUCCA motif on HeLa mRNA did not show enrichment at the 5′-end of the transcript. **m**) In contrast to Fig. 5l, the detected m^5^C sites within the CUCCA motif on HEK293T mRNA did not show enrichment at the 5′ end of the transcript.

The majority of the detected m^5^C sites displayed 5–20% modification fractions, but one-fourth of sites (768 and 604 sites, respectively) still showed fractions ≥20% and one-fifth (517 and 403 sites) showed fractions ≥33% (Fig. 5a,b). The types of m^5^C-modified genes are similar between the two cell lines. About 80% of the detected m^5^C sites are in the protein-coding region (Extended Data Fig. 9a,b), among which more than half of the sites are in the coding sequence (CDS) region (Fig. 5c,d). The two cell lines also consistently exhibited only one m^5^C site among most of the m^5^C-modified genes (Fig. 5e,f).

Although the overall m^5^C modification patterns of the two cell lines are similar, differences were also observed (Extended Data Fig. 9c). m^5^C sites in mRNA from the HEK293T cells exhibited twofold to threefold higher enrichment in the CUCCA motif compared with those from HeLa cells (Fig. 5g,h). In HEK293T cells 40% of mRNA m^5^C sites are in the CUCCA motif, while the number for HeLa cells is only 14%. Excluding the CUCCA motif, the remaining motifs exhibit a G-rich pattern. CUCCA motif was reported as the substrate of NSUN6 (ref. 10), while the G-rich motifs were reported to be substrates of NSUN2 (ref. 9). Furthermore, our data revealed that m^5^C sites on the CUCCA motif tend to have a higher modification fraction in HEK293T cells compared to HeLa cells. Conversely, m^5^C sites on G-rich motifs exhibited a higher modification level in HeLa cells than those in HEK293T cells (Extended Data Fig. 9d,e). The high enrichment of CUCCA motifs in HEK293T cells suggested that the NSUN6 methyltransferase has a more notable role in m^5^C installation in HEK293T cells, while in HeLa cells, NSUN2 is the primary methyltransferase responsible for m^5^C deposition (Extended Data Fig. 9f).

**Extended Data Fig. 10 ∣:**
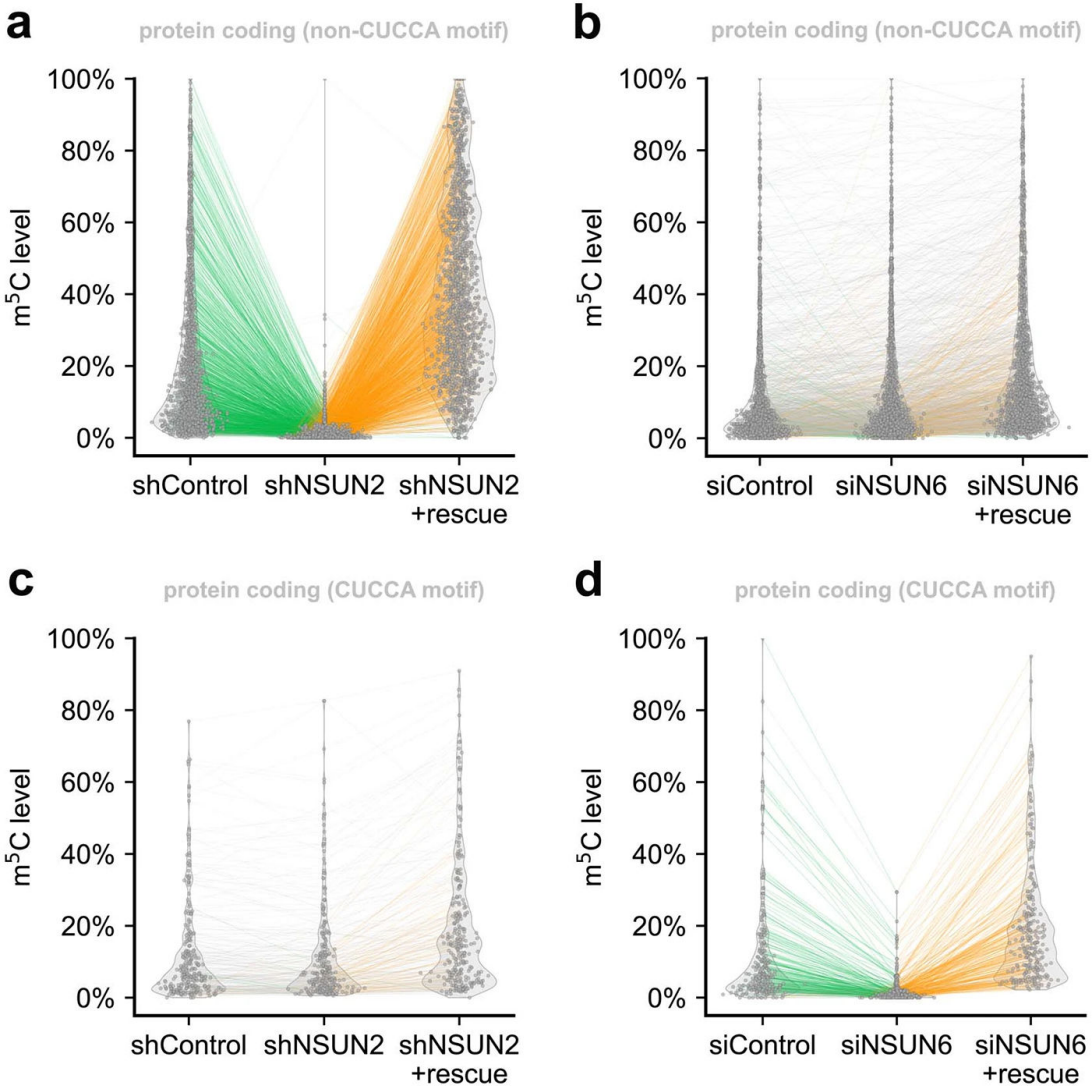
Rescue of the detected m^5^C sites in NSUN2 or NSUN6 depletion. **a**) m^5^C sites with non-CUCCA motifs under NSUN2 knockdown and rescue experiment. **b**) m^5^C sites with non-CUCCA motifs under NSUN6 knockdown and rescue experiment. **c**) m^5^C sites with CUCCA motifs under NSUN2 knockdown and rescue experiment. **d**) m^5^C sites with CUCCA motifs under NSUN6 knockdown and rescue experiment.

To further validate the detected m^5^C sites in these motifs and the corresponding NSUN assignments, we knocked down *NSUN2* in HeLa cell line (Extended Data Fig. 9g) and found that the modification fraction of ~90% of the m^5^C sites, mostly within G-rich motifs, substantially decreased (Fig. 5i, Extended Data Fig. 9i and Supplementary Table 8), confirming that UBS-seq generates a very low false-positive rate. Similarly, m^5^C sites on CUCCA motifs were also validated in *NSUN6* knockdown HeLa cells (Fig. 5j, Extended Data Fig. 9h,j and Supplementary Table 9). Results from knockdown experiments were consistent with the finding^8,10^ that NSUN2 and NSUN6 are the two main m^5^C writer proteins for mammalian mRNA m^5^C deposition and they have different sequence preferences (Extended Data Fig. 9k). To further validate the detected m^5^C sites with low modification fractions, we conducted rescue experiments by transfecting *NSUN2-* or *NSUN6-*containing plasmids back to the HeLa cells and sequenced the isolated poly(A)-tailed RNA. Indeed, we observed that the decreased m^5^C fractions in the depleted strains were mostly rescued, further confirming that these lowly modified m^5^C sites are real (Extended Data Fig. 10).

The conventional criterion for calling a modified m^5^C site is to set a certain unconverted rate. Accordingly, we chose >5% stoichiometry as the cutoff for the detected m^5^C sites (Fig. 4a,b). However, by doing so some m^5^C sites could be missed due to low gene expression level, and many real m^5^C sites with <5% stoichiometry could be neglected as well. We have several reasons to believe that the m^5^C sites with <5% stoichiometry may not be false positives, which are as follows: (1) our UBS-seq gave much higher BS efficiency and much lower background, with the statistical method we are confident that many of these sites could be real m^5^C sites; (2) these sites show similar motifs as those with >5% stoichiometry; (3) the detected stoichiometries of these sites are also sensitive to *NSUN2* or *NUSN6* knockdown; (4) these sites displayed a similar 5′-end enrichment pattern as those with >5% stoichiometry; and (5) the lower m^5^C stoichiometry detected in mRNA with *NSUN2* or *NUSN6* knockdown could be rescued after *NSUN2* or *NUSN6* plasmids were transfected back to HeLa cells.

The functional significance of these lowly modified m^5^C sites remains to be investigated. The presence of these low stoichiometry m^5^C sites may suggest that (1) m^5^C might have quite variable distribution at the single-cell level and may show high cell heterogeneity, and (2) m^5^C could be only installed in some specific RNAs in certain cellular granules. It is also possible that many of these sites are moonlight activities from methyltransferases. It would be interesting to develop methods to allow m^5^C mapping in the single-cell level or in different RNA granules in the future.

Interestingly, both HeLa and HEK293T showed a similar enrichment pattern at the 5′-end of transcripts for m^5^C sites deposited by NSUN2 (Fig. 5k,l) but not those deposited by NSUN6 (Extended Data Fig. 9l,m). In connection with ribosome profiling data^36^, we found evidence that m^5^C modification at the 5′-end of transcripts may modulate translation efficiency^37^. Genes with m^5^C sites at 5′-end enrich more ribosomal signals at the 5′-UTR of the transcripts when compared to nonmethylated genes (*P* = 1.05 × 10^−6^), whereas genes with m^5^C sites at 3′-end do not significantly exhibit an enrichment signal of the ribosome (*P* = 0.37). Additionally, m^5^C methylated genes do not exhibit enrichment signals in the CDS region either (Fig. 5m).

## Discussion

In high eukaryotes, 5mC in gDNA is the most abundant and important epigenetic mark. The intense interest in its biological function and its role in human diseases have led to the development of numerous methods to detect DNA methylation^6,7^. Among them, BS-seq remains the gold standard and has been widely used in basic research and clinical applications. Despite its success, conventional BS-seq could be improved from several limitations, including a lengthy reaction time, severe DNA damage, incomplete C-to-U conversion at high GC regions, uneven and missing coverage, biased representation of methylated versus unmethylated DNA due to biased DNA cleavage sites and incomplete deamination of 4mC. These limitations present bigger challenges when performing 5mC detection in low-input samples without DNA purification. Based on the mechanistic insight of C-to-U conversion and DNA degradation mediated by BS treatment, we addressed all these drawbacks by using ammonium instead of sodium salt of bisulfite to achieve a much higher bisulfite concentration and higher reaction temperature to accelerate the reaction and denature DNA/RNA so that BS conversion can complete within several minutes. Our UBS-1 condition not only improves the BS efficiency to notably reduce background, especially at highly structured DNA regions, but also reduces DNA degradation and minimizes the overestimation of 5mC level. In addition, the 4mC-to-U conversion rate becomes quantitative in UBS-seq, avoiding potential false positives caused by the 4mC present in certain gDNA.

When applying UBS-seq to mESC gDNA, we found that UBS-seq consistently afforded much lower backgrounds and smaller proportions of non-CpG motifs than conventional BS-seq, indicating a lower false-positive rate for UBS-seq. In addition, UBS-seq significantly reduced the unconverted ratio at all GC content regions compared with the conventional BS-seq condition and consistently showed less degradation on gDNA, resulting in more evenly distributed genome coverage. In contrast, conventional BS-seq showed systematically higher methylation levels among all the 100 kb genomic bins than UBS-seq, suggesting that the more serious DNA degradation in conventional BS-seq could result in overestimation of the methylation level in the whole genome. These observations validated that UBS-seq outperforms conventional BS-seq in terms of lower background, higher CpG and genome coverage, less overestimation of 5mC fraction and more accurate at characterizing 5mC at the non-CpG sites.

When applying UBS-seq and conventional BS-seq directly to a small number of cells, we found that background was a much more severe problem for 5mC detection at single cell to 100-cell level for conventional BS-seq. UBS-seq performed better with ~20-fold lower background than conventional BS-seq. Further analysis on mtDNA in these libraries revealed that the background in mtDNA in all libraries is much higher than that in λ-DNA spike-in, due to the presence of highly structured regions in mtDNA. Once again, UBS-seq showed much lower background noise, further demonstrating its superior performance over conventional BS-seq. Our results also suggest that UBS-seq is more suitable for 5mC sequencing in cfDNA.

In addition to detecting DNA methylation, the optimized UBS-seq solves a critical technology challenge for RNA m^5^C sequencing. In conventional RNA m^5^C BS-seq, the major issue has been the high false-positive rates caused by incompletion C-to-U conversion due to reduced reaction temperature and time to avoid severe RNA degradation. The reduced temperature is also ineffective in denaturing local secondary structures of highly structured RNAs, leading to further reduced C-to-U conversion at structured regions. We show that UBS-seq for RNA was able to effectively remove the false positives without compromising accurate m^5^C detection and quantification. As little as 10–20 ng mRNA could be used with thousands of confident m^5^C sites detected in HeLa and HEK293T mRNAs. The quantitative nature of UBS-seq allowed us to reveal sequence motifs of m^5^C sites in mRNA and assign NSUN2 as the major m^5^C methyltransferase that installs ~90% of m^5^C sites to HeLa mRNA. In addition, our results showed that m^5^C sites deposited by NSUN2 but not NSUN6 are enriched in 5′-UTR regions in both HeLa and HEK293T mRNA, suggesting that m^5^C modification or its binding proteins may be involved in regulating mRNA translation. This method and the datasets will greatly aid future functional investigations on RNA m^5^C.

### Limitations of the study

UBS-seq still shares two drawbacks of conventional BS-seq. One is low complexity of the reads caused by the C-to-U conversion, which may cause mapping issues. This is the inherent problem associated with the deamination methods in general. However, we can minimize the problem by constructing libraries with longer fragments and by sequencing the libraries using pair-end to improve the mapping ratio. With further improvement in data analysis, the mapping issue caused by the low complexity could be minimized. The other is that 5mC cannot be distinguished from 5-hydroxymethylcytosine (5hmC) in BS-seq. BS treatment converts 5hmC to 5-cytosinemethylenesulfonate (CMS) that cannot proceed to deamination, therefore both 5mC and 5hmC are to be read as C and cannot be distinguishable by BS-seq only. Because the numbers of 5hmC sites in most mammalian genomes are much smaller compared with those of 5mC, this is usually not a problem. However, when it is necessary to distinguish 5mC sites from 5hmC, a further step of APOBEC3A enzyme treatment can be added. It has been reported that APOBEC3A treatment can efficiently deaminate 5mC but only partially of 5hmC^2^, but CMS-modified 5hmC completely resists the APOBEC3A-mediated deamination. Therefore, we reason that after UBS-seq, we obtain the sum of 5mC and 5hmC. A further treatment with APOBEC3A would deaminate 5mC leaving only 5hmC sites that can be read as C.

## Methods

### Cell culture

HeLa, HEK293T and A549 cells were purchased from the American Type Culture Collection. Cells were cultured at 37 °C with 5.0% CO_2_ in a Heracell VIOS 160i incubator (Thermo Fisher Scientific). All cell lines were grown in DMEM medium (Gibco, 11995) supplemented with 10% vol/vol FBS and 1% penicillin/streptomycin (Gibco). The percentage of surviving cells after treatment was assessed by the sulforhodamine B (SRB) assay. Cell cycle distribution and cell size determination were assessed by flow cytometry. mES cell line was cultured at 37 °C with 5.0% CO_2_ on the 6-cm dishes coated with 0.2% gelatin (Gibco) and the layer of mouse feeder cells (Gibco). Cell culture was in complete DMEM medium supplemented with 15% vol/vol FBS, 1% penicillin/streptomycin, 1.25× nucleoside (MilliporeSigma), 62.5 mM 2-mercaptoethanol (Thermo Fisher Scientific), 1.25× nonessential amino acids (Gibco), 104 units per ml leukemia inhibitory factor (LIF) (MilliporeSigma), 0.289 mg per 500 ml of PD0325901 (STEMCELL Technologies), 0.83 mg per 500 ml of CHIR99021 (STEMCELL Technologies), 5 mg ml^−1^ of Plasmocin Prophylactic (Invitrogen).

### RNA isolation

1. Total RNA isolation: cellular total RNA was isolated from the cells using TRIzol reagent (Ambion by Life Technologies) and Direct-zol RNA miniprep kit (Zymo Research) following the manufacturer’s protocol. In brief, cells from 10-cm plates were suspended in 1 ml of TRIzol reagent, centrifuged at 16,000*g* for 1 min and the supernatant was mixed with one volume of 100% ethanol. RNA was bound to the column and treated with DNase I at room temperature for 15 min. RNA was washed with washing buffer and eluted with 50 μl RNase-free water.
2. polyA-tailed RNA isolation: two rounds of poly(A) enrichment were conducted using DynaBeads mRNA Direct Purification Kit (Thermo Fisher Scientific) following the manufacturer’s protocol with some modifications. In brief, 200 ml of Dynabeads were washed with 200 ml of lysis/binding buffer and mixed with 100 μg of total RNA in 300 ml of lysis/binding buffer. Samples were incubated at 65 °C for 2 min, followed by incubation on the roller mixer at room temperature for 20 min. Then the beads were washed twice with washing buffer A and once with washing buffer B in the kit. Beads were resuspended in 50 μl of 10 mM Tris–HCl (pH 7) and incubated at 70 °C for 3 min for washing and then eluted with 10 μl of buffer. Eluate was then used for the second round of polyA enrichment in the same procedure as described above.

### DNA isolation

#### gDNA isolation.

DNA was extracted using a Quick DNA/RNA Miniprep Kit (Zymo Research) following the manufacturer’s protocol. In brief, cells were collected from 10-cm plates and lysed with DNA/RNA lysis buffer. The lysate was transferred to Spin-Away Filter and centrifuged at 16,000*g* for 30 s, and the column was washed with DNA/RNA Prep Buffer, followed by two washes with DNA/RNA Wash Buffer. DNA was eluted with H_2_O.

#### siRNA, shRNA knockdown and *NSUN2* KO.

For transient transfection, cells were transfected with siRNA from Qiagen (siCtrl: SI03650318, siN-SUN6: SI00162659) by Lipofectamine RNAiMAX Transfection Reagent (Invitrogen) following commercial protocols. For lentivirus production, pLKO-Tet-On (shCtrl: ATCTCGCTTGGGCGAGAGTAAG, shNSUN2: GAGCGATGCCTTAGGATATTA) together with pMD2.G (Addgene, 12259) and psPAX2 (Addgene, 12260) were cotransfected into 293TN cells (System Biosciences). Viruses were concentrated by the PEG-it Virus Precipitation Solution and used for infecting HeLa cells in the presence of TransDux (System Biosciences). Transfected cells were selected by 2 μg ml^−1^ puromycin. Pools of stable transfectants were selected by antibiotics or sorted by flow cytometry. Doxycycline (1 μg ml^−1^) was used to induce shRNA. For the *NSUN2* KO A549 cells, cell lines were produced as described previously^38^.

### Model DNA and RNA oligo synthesis

Unmodified and modified DNA and RNA oligos were synthesized in-house using Expedite DNA synthesizer. Unmodified phosphoramidites and 5-methylcytosine phosphoramidites for DNA (5-Me-dC-CE phosphoramidite, 10-1060-90) or RNA (5-Me-C-TOM-CE phosphoramidite, 10-3064-95) and other reagents for oligo synthesis were purchased from Glen Research. DNA containing 4mC modification was synthesized using a convertible *O*^4^-triazolyl-dU-CE phosphoramidite (10-1051-90). After oligo synthesis, 40% methylamine in water was used for deprotection and converting *O*^4^-triazolyl-dU to 4mC.

### Reaction of model DNA/RNA oligonucleotides with UBS reagents and monitoring of the reaction by MALDI-TOF MS

In total, 9 μl of the BS reagent was preheated at 98 °C for 5 min, and then 1 μl of 5-mer DNA or RNA oligo (50 ng μl^−1^) was added and the mixture was further incubated at 98 °C for 1–10 min. After cooling to room temperature, 2 μl of the reaction mixture was added to 40 μl resin (Bio-Rad) and allowed to stand at room temperature for 30 min. Then 1.8 μl supernatant was mixed with matrix 2′,4′,6′-trihydroxyacetophenone monohydrate and loaded onto a MALDI plate. The MALDI-TOF MS was recorded on an Ultra-flex TOF/TOF MALDI mass spectrometer using the negative ion reflection mode (Bruker). Data were processed in Flex Analysis software (Bruker).

### DNA degradation test

One of the 50 ng of HeLa gDNA samples was used as the input sample, the sample treated by UBS-1 and the control sample treated with the conventional BS condition following the manufacturer’s protocol (DNA Methylation-Gold Kit; Zymo Research), respectively. Ultrafast bisulfite reagent was added to DNA in a 9:1 ratio (vol/vol), and the reaction was incubated at 98 °C for 10 min, followed by desulphonation on Zymo-Spin IC columns following the manufacturer’s protocol except the final washing step was repeated for four times instead of twice as described in Zymo kit. Samples were eluted in 20 μl of water and mixed with 20 μl of Novex Tris-borate-EDTA (TBE)-Urea Sample Buffer (2×; Invitrogen) to run on 10% Novex TBE-Urea Gel (Invitrogen) at constant 180 V for 80 min. The gel was stained with 2 μl of SYBR Gold Nucleic Acid Gel Stain (Invitrogen) in about 25 ml of TBE buffer at room temperature for 15 min. The gel was then illuminated using the ChemiDoc MP imaging system (Bio-Rad) at the University of Chicago BioPhysics Core Facility.

### RNA degradation test

One of the 50 ng of HeLa total samples was used as the input sample and one was treated by UBS-2, and the other two samples were treated with the reported BS conditions^8^, respectively. UBS-2 bisulfite reagent was added to DNA in a 9:1 ratio (vol/vol), and the reaction was incubated at 98 °C for 10 min, followed by desulphonation on Zymo-Spin IC columns following the manufacturer’s protocol except the final washing step was repeated for four times instead of twice as described in Zymo kit. Samples were eluted with 20 μl of water and mixed with 20 μl of Novex TBE-Urea Sample Buffer (2×; Invitrogen) to run on 10% Novex TBE-Urea Gel (Invitrogen) at constant 180 V for 80 min. The gel was stained with 2 μl of SYBR Gold Nucleic Acid Gel Stain (Invitrogen) in about 25 ml of TBE buffer at room temperature for 15 min. The gel was then illuminated using the ChemiDoc MP imaging system (Bio-Rad) at the University of Chicago BioPhysics Core Facility.

### Sanger sequencing

The 100 bp DNA oligo containing 5mC and 4mC sites (sequence in Supplementary Table 1) was treated with UBS-1 and BS reagent in DNA Methylation-Gold Kit (Zymo Research) side by side. After treatment, the DNA was purified with DNA Clean and Concentrate Kit (Zymo Research). Primers matching the 5′ and 3′ flanking regions of the oligo were used for a ten round of PCR amplification, and the PCR product was used for Sanger sequencing with reverse primer.

### Library preparation

#### Library preparation starting from mES gDNA.

The mixture of mES gDNA (100–200 ng μl^−1^) and spike-in λ-DNA (0.1%) was fragmented by sonication in a 100 μl tube for 11 cycles of 30 s on and 30 s off using Bioruptor Pico (Diagenode). The fragmented gDNA was size selected by Agencourt AMPure XP (Beckman Coulter) to 300–500 bp and eluted with 17 μl of water. After end repairing and A-tagging, the DNA samples were ligated to a methylated adaptor using NEBNext Ultra II DNA Library Prep Kit (NEB, E7103) and purified by beads in a 0.9× ratio. The DNA samples were then divided into three groups, with one group as the untreated input and the other two groups further divided so that each group contained 10 ng and 1 ng samples in triplicates, respectively. One group of the samples was treated with CMS-1 in a 1:9 ratio (vol/vol) at 98 °C for 10 min, while the other group of samples was treated with the BS reagent in the DNA Methylation-Gold Kit (Zymo Research) following the manufacturer’s protocol side by side. After BS treatment, all samples were desulphonated on Zymo-Spin IC columns following the manufacturer’s protocol except that the final washing step was conducted four times instead of twice. qPCR was performed to quantify the number of DNA copies. Based on qPCR results, PCR was performed using 2× LongAmp Taq mix, universal and index primers (NEB). DNA was purified in two rounds with 0.6× AMPure XP beads. The quality of libraries was examined by Bioanalyzer, and the libraries were sequenced on the Illumina Nova-Seq 6000 platform.

#### Library preparation starting from a small number of mES cells (100 cell, 10 cell and single cell).

The DNA libraries starting from a small number of mES cells were constructed following the previous method with slight modifications^39,40^. In brief, mES cells were sorted by fluorescence-activated cell sorting into lysis buffer containing 20 mM Tris–HCl (pH 8.0), 2 mM EDTA, 20 mM KCl, 0.3% Triton-X 100, 1 mg ml^−1^ QIAGEN protease and 1% of λ-DNA (dam^−^, dcm^−^), which is 0.06, 0.6 and 6 pg for single cell, 10 and 100 cells, respectively. gDNA was released under the program of 50 °C for 3 h, followed by 75 °C for 30 min. Bisulfite treatment was performed using UBS-1 and Zymo DNA Methylation-Gold Kit side by side. The first-strand DNA was synthesized by random priming using 50 U Klenow exo^−^, 400 μM P5-N6-oligo1 (CTACACGACGCTCTTCCGATCTNNNNNN, IDT) at 37 °C for 30 min. After four rounds of random priming, samples were purified with 0.8× AMPure XP beads. The second strand was synthesized using P7-N6-oligo2 (AGACGTGTGCTCTTCCGATCTNNNNNN, IDT). After purification with 0.8× AMPure XP beads, the sequencing index was introduced by PCR using Multiplex Oligos for Illumina (NEB) and KAPA HiFi HotStart ReadyMix.

#### Library preparation for human plasma cfDNA.

In total, 41 ng of plasma cfDNA either from healthy people or from a patient’s sample was spiked-in with 0.1% of the 164 bp dsDNA containing four 5mC sites, and water was added to give each sample a total volume of 25 μl. Then to each sample, End Repair and A-Tailing Buffer (3.5 μl) and End Repair and A-Tailing Enzyme Mix from NEBNext Ultra II DNA Library Prep Kit (NEB, E7103) were added and the mixture was incubated at 65 °C for 30 min. Then a mixture of 15 μl of ligation buffer, 5 μl of DNA ligase, 2.5 μl of water and 2.5 μl of 15 μM methylated NEBNext adaptor was added, and the mixture was incubated at 20 °C for 1 h. Then 3 μl of USER enzyme (NEB, M5505S) was added and incubated at 37 °C for 15 min. The ligated DNA was purified by 1.0× Ampure beads, eluted with 41 μl of water and divided into five parts. One part equivalent to 1 ng cfDNA was directly amplified as input library. Two parts containing 10 ng cfDNA were treated with UBS-1 condition followed by desulphonation following the same procedure as described above for mES gDNA, and the other two parts equivalent to 10 ng cfDNA were treated with the conventional BS condition following the manufacturer’s protocol. The purified DNA was then amplified using KAPA HiFi Uracil^+^ DNA polymerase (Roche, 07959079001), and the libraries were purified by 0.7× Ampure beads.

### Library preparation for RNA

1. Bisulfite treatment: Culture of WT HeLa, HEK293T or A549 cells; HeLa shControl or NSUN2 knockdown cells and polyA^+^ RNA purification was conducted as described. In total, 45 μl of UBS-2 reagent was preheated to 98 °C for 5 min in a PCR instrument, and then around 20 ng RNA in 5 μl of water was added and mixed well. The mixture was incubated at 98 °C for 9 min with the lid temperature set as 105 °C. After cooling to room temperature, desulphonation was accomplished on the Zymo-Spin IC columns following the manufacturer’s protocol. To the sample was added 100 μl of H_2_O, 250 μl of binding buffer (BB) buffer and 400 μl of 100% EtOH. Next, the sample was then centrifuged for 30 s at 16,000*g*. Again, 200 μl of washing buffer was added and the sample was centrifuged. Again 200 μl of desulphonation buffer was added, and the sample was incubated at room temperature for 75 min and centrifuged. The sample was washed twice with 400 μl of wash buffer, discard flow through. Again centrifuged for an additional 2 min to get rid of all the buffer and finally eluted with 34.84 μl of H_2_O.
2. Alkaline fragmentation: To the BS-treated RNA or untreated RNA for input libraries in 34.84 μl of H_2_O was added 3.76 μl NaHCO_3_ (pH 9.2). The mixture was incubated at 95 °C for 3 min for BS-treated samples and 4 min at 95 °C for input samples. After cooling to room temperature, NaOAc (1 μl, 3 M, pH 5.2) was added to adjust the pH of the reaction mixture to neutral.
3. 3′-Repair and 5′-phosphorylation: After alkaline fragmentation and neutralization, the reaction mixture (38.6 μl) was supplemented with 4.4 μl of 10x T4 PNK buffer (NEB, B0201S) and 1 μl of T4 PNK (NEB), and the mixture was incubated at 37 °C for 30 min; then 0.5 μl of T4 PNK and 5 μl of 10 mM ATP were added for another incubation at 37 °C for 30 min, followed by RNA Clean and Concentrator (Zymo Research) purification eluting with 6 μl of RNase-free water.
4. 3′-Adapter ligation: The 3′-repaired and 5′-phosphorylated RNA fragments were incubated with 1 μl of RNA 3′-SR Adapter (5′-App-NNNNNATCACGAGATCGGAAGAGCACA CGTCT-3SpC3, with ATCACG as the inline barcode) at 70 °C for 2 min and placed immediately on ice. Then 10 μl of 3′-ligation buffer and 3 μl of enzyme mix from NEB small RNA library kit (E7330L) were added and the mixture was incubated at 16 °C overnight. The excessive adapters were digested by adding 1 μl of 5′-deadenylase (NEB, M0331S) at 30 °C for 30 min followed by adding 1 μl of RecJf (NEB, M0264L) at 37 °C for 30 min. The 3′-end-ligated RNA was purified by RNA Clean and Concentrator (Zymo Research) and eluted with 10 μl of RNase-free water.
5. SR RT primer annealing: Then 1 μl of 5.625 μM 3′-SR RT primer (5′-AGACGTGTGCTCTTCCGATCT-3′) was added to ligated RNA and further incubated in a thermocycler for 5 min at 75 °C, 15 min at 37 °C and 15 min at 25 °C.
6. 5′-Adapter ligation: The annealed RNA was incubated with 1 μl of 5.625 μM 5′-SR Adapter (5′-GUUCAGAGUUCUACAGUCCGACGAUC NNNNN-3′) at 70 °C for 2 min and placed immediately on ice. Then 1 μl of 5′-ligation buffer and 2.5 μl of 5′-ligation enzyme mix were added, and the mixture was incubated at 25 °C for 16 h.
7. RT reaction: A total of 4 μl of RT reaction buffer (5×), 1 μl of murine RNase inhibitor and 1 μl of ProtoScript II reverse transcriptase were added to the ligated RNA. The mixture was incubated at 50 °C for 60 min and 70 °C for 15 min. After RT, qPCR was performed to quantify the number of cDNA copies.
8. PCR amplification and sequencing: Based on qPCR results, PCR was performed using 2× LongAmp Taq mix (NEB) and SR and index primers (NEB). The libraries were purified by running a 2% low melting agarose gel, and the desired bands were cut. The library DNA was extracted from the gel using the MinElute Gel Extraction Kit (Qiagen) following the manufacturer’s protocol. The quality of the libraries was checked on Bioanalyzer, and the libraries were sequenced on the Illumina Nova-Seq 6000 platform with single-end 100 bp read length.

### NGS sequencing data processing and analysis

#### DNA 5mC profiling.

After trimming the TruSeq sequencing adapters from the 3′ ends of read 1 and read 2 using the cutadapt tool, low-quality and short reads were filtered out by applying ‘-q 20 –nextseq-trim 20 –max-n 0 -m 20’ arguments. The clean reads were then aligned to the spike-in sequences (the 164 bp synthetic dsDNA oligo in Supplementary Table 1 and GenBank J02459.1 for λ-DNA) and reference genomes (mouse reference GRCm39 for mESC samples and human reference GRCh38 for cfDNA samples) using the hisat-3n tool. The ‘--base-change C,T --no-splice-alignment –bowtie2-dp 1 –score-min L,0,1’ argument was used for all DNA libraries to ensure accurate alignment. To make use of the strand-specific property of the library, the ‘–directional-mapping’ parameter was applied for 10 ng/1 ng mES gDNA and cfDNA libraries, while ‘--directional-mapping-reverse’ parameter was used for single-cell mESC libraries. In this context, read one aligns solely with either the converted reference sequence or its reverse complement, rather than indiscriminately aligning with both. The sorted mapped reads were then processed to remove PCR duplicates using the MarkDuplicateSpark command in the GATK tool. Finally, the number of converted and unconverted reads at all C sites was counted using the hisat-3n-table command, and the methylation ratio was calculated as the number of unconverted reads divided by total coverage (Extended Data Fig. 10).

#### RNA m^5^C profiling.

Like the DNA 5mC libraries, the adapter sequence and low-quality reads were trimmed from the RNA m^5^C libraries using the cutadapt tool. Only reads with the correct inline barcode (ATCACG) were retained, and 5 nt of the UMI at the 5′-end of the insert plus 5 nt of the UMI at the 3′-end of the insert fragments were extracted. Clean reads were mapped to the corresponding reference sequence using the hisat-3n tool^41^. To reduce mapping bias, reads were first mapped to rRNA and tRNA genes, which have multiple copies in the genome and are highly expressed, and filtered reads were then mapped to the reference genome (GRCh38) using hisat-3n with the same setting as DNA 5mC data analysis, expect the ‘--no-splice-alignment’ argument. To remove mapping errors, reads with more than 5% of mismatches (not including C to T conversions) in the mapping were discarded. To eliminate unconverted clusters, reads with more than three unconverted C sites or reads with more than one-third of the total C sites being unconverted were also discarded. Background noise was estimated from all the C sites within each library, respectively, and a binomial model as described in Supplementary Note was used to calculate a *P* value for each site. Sites with a *P* value less than 10^−6^ were classified as m^5^C sites, indicating a significant level of unconverted reads that was unlikely to be due to background noise.

### Statistics and reproducibility

For UBS-seq libraries, one or two technical replicate(s) were used in each experiment with cultured cells.

## Extended Data

## Supplementary Material

Sup Info

Sup Table 1

Sup Table 2

Sup Table 3

Sup Table 4

Sup Table 5

Sup Table 6

Sup Table 7

Sup Table 8

Sup Table 9

Sup Table 10

## Data Availability

Data are available at the Gene Expression Omnibus under accession GSE225614 (ref. 42). For benchmarking, we used the following accessions: GSE93751, GSE122260 and GSE151028. Source data are provided with this paper.

## Code availability

Mapping and analysis scripts are available at https://github.com/y9c/m5C-UBSseq (ref. 43).

## References

[1] Dor Y & Cedar H Principles of DNA methylation and their implications for biology and medicine. Lancet 392, 777–786 (2018). 30100054 10.1016/S0140-6736(18)31268-6

[2] Loyfer N. A DNA methylation atlas of normal human cell types. Nature 613, 355–364 (2023). 36599988 10.1038/s41586-022-05580-6 PMC9811898

[3] Frommer M. A genomic sequencing protocol that yields a positive display of 5-methylcytosine residues in individual DNA strands. Proc. Natl Acad. Sci. USA 89, 1827–1831 (1992). 1542678 10.1073/pnas.89.5.1827 PMC48546

[4] Fraga MF & Esteller M DNA methylation: a profile of methods and applications. Biotechniques 33, 632–649 (2002). 12238773 10.2144/02333rv01

[5] Olova N. Comparison of whole-genome bisulfite sequencing library preparation strategies identifies sources of biases affecting DNA methylation data. Genome Biol. 19, 33 (2018). 29544553 10.1186/s13059-018-1408-2 PMC5856372

[6] Vaisvila R. Enzymatic methyl sequencing detects DNA methylation at single-base resolution from picograms of DNA. Genome Res. 31, 1280–1289 (2021). 34140313 10.1101/gr.266551.120 PMC8256858

[7] Liu Y. Bisulfite-free direct detection of 5-methylcytosine and 5-hydroxymethylcytosine at base resolution. Nat. Biotechnol 37, 424–429 (2019). 30804537 10.1038/s41587-019-0041-2

[8] Yang X. 5-Methylcytosine promotes mRNA export—NSUN2 as the methyltransferase and ALYREF as an m5C reader. Cell Res. 27, 606–625 (2017). 28418038 10.1038/cr.2017.55 PMC5594206

[9] Huang T, Chen W, Liu J, Gu N & Zhang R Genome-wide identification of mRNA 5-methylcytosine in mammals. Nat. Struct. Mol. Biol 26, 380–388 (2019). 31061524 10.1038/s41594-019-0218-x

[10] Selmi T. Sequence- and structure-specific cytosine-5 mRNA methylation by NSUN6. Nucleic Acids Res. 49, 1006–1022 (2021). 33330931 10.1093/nar/gkaa1193 PMC7826283

[11] Hussain S, Aleksic J, Blanco S, Dietmann S & Frye M Characterizing 5-methylcytosine in the mammalian epitranscriptome. Genome Biol. 14, 215 (2013). 24286375 10.1186/gb4143 PMC4053770

[12] Chen X. 5-Methylcytosine promotes pathogenesis of bladder cancer through stabilizing mRNAs. Nat. Cell Biol 21, 978–990 (2019). 31358969 10.1038/s41556-019-0361-y

[13] Nishida N. Aberrant methylation of multiple tumor suppressor genes in aging liver, chronic hepatitis, and hepatocellular carcinoma. Hepatology 47, 908–918 (2008). 18161048 10.1002/hep.22110 PMC2865182

[14] Cheray M. Cytosine methylation of mature microRNAs inhibits their functions and is associated with poor prognosis in glioblastoma multiforme. Mol. Cancer 19, 36 (2020). 32098627 10.1186/s12943-020-01155-z PMC7041276

[15] Gaiti F. Epigenetic evolution and lineage histories of chronic lymphocytic leukaemia. Nature 569, 576–580 (2019). 31092926 10.1038/s41586-019-1198-z PMC6533116

[16] Cui X. 5-Methylcytosine RNA methylation in *Arabidopsis thaliana*. Mol. Plant 10, 1387–1399 (2017). 28965832 10.1016/j.molp.2017.09.013

[17] Khoddami V & Cairns BR Identification of direct targets and modified bases of RNA cytosine methyltransferases. Nat. Biotechnol 31, 458–464 (2013). 23604283 10.1038/nbt.2566 PMC3791587

[18] Schaefer M, Pollex T, Hanna K & Lyko F RNA cytosine methylation analysis by bisulfite sequencing. Nucleic Acids Res. 37, e12 (2009). 19059995 10.1093/nar/gkn954 PMC2632927

[19] Janin M. Epigenetic loss of RNA-methyltransferase NSUN5 in glioma targets ribosomes to drive a stress adaptive translational program. Acta Neuropathol. 138, 1053–1074 (2019). 31428936 10.1007/s00401-019-02062-4 PMC6851045

[20] Blanco S. Aberrant methylation of tRNAs links cellular stress to neuro-developmental disorders. EMBO J. 33, 2020–2039 (2014). 25063673 10.15252/embj.201489282 PMC4195770

[21] Squires JE Widespread occurrence of 5-methylcytosine in human coding and non-coding RNA. Nucleic Acids Res. 40, 5023–5033 (2012). 22344696 10.1093/nar/gks144 PMC3367185

[22] Legrand C. Statistically robust methylation calling for whole-transcriptome bisulfite sequencing reveals distinct methylation patterns for mouse RNAs. Genome Res. 27, 1589–1596 (2017). 28684555 10.1101/gr.210666.116 PMC5580717

[23] Tanaka K & Okamoto A Degradation of DNA by bisulfite treatment. Bioorg. Med. Chem. Lett 17, 1912–1915 (2007). 17276678 10.1016/j.bmcl.2007.01.040

[24] Shapiro R, DiFate V & Welcher M Deamination of cytosine derivatives by bisulfite. Mechanism of the reaction. J. Am. Chem. Soc 96, 906–912 (1974). 4814744 10.1021/ja00810a043

[25] Sono M, Wataya Y & Hayatsu H Role of bisulfite in the deamination and the hydrogen isotope exchange of cytidylic acid. J. Am. Chem. Soc 95, 4745–4749 (1973). 4730665 10.1021/ja00795a044

[26] Hayatsu H, Negishi K & Shiraishi M DNA methylation analysis: speedup of bisulfite-mediated deamination of cytosine in the genomic sequencing procedure. Proc. Jpn. Acad. Ser. B Phys. Biol. Sci 80, 189–194 (2004).

[27] Shiraishi M & Hayatsu H High-speed conversion of cytosine to uracil in bisulfite genomic sequencing analysis of DNA methylation. DNA Res. 11, 409–415 (2004). 15871463 10.1093/dnares/11.6.409

[28] Grunau C, Clark SJ & Rosenthal A Bisulfite genomic sequencing: systematic investigation of critical experimental parameters. Nucleic Acids Res. 29, e65 (2001). 11433041 10.1093/nar/29.13.e65 PMC55789

[29] Rodriguez F, Yushenova IA, DiCorpo D & Arkhipova IR Bacterial N4-methylcytosine as an epigenetic mark in eukaryotic DNA. Nat. Commun 13, 1072 (2022). 35228526 10.1038/s41467-022-28471-w PMC8885841

[30] Yu M. Base-resolution detection of N4-methylcytosine in genomic DNA using 4mC-Tet-assisted-bisulfite-sequencing. Nucleic Acids Res. 43, e148 (2015). 26184871 10.1093/nar/gkv738 PMC4666385

[31] De Mendoza A. The emergence of the brain non-CpG methylation system in vertebrates. Nat. Ecol. Evol 5, 369–378 (2021). 33462491 10.1038/s41559-020-01371-2 PMC7116863

[32] Liu H. DNA methylation atlas of the mouse brain at single-cell resolution. Nature 598, 120–128 (2021). 34616061 10.1038/s41586-020-03182-8 PMC8494641

[33] Ding SC & Lo YMD Cell-free DNA fragmentomics in liquid biopsy. Diagnostics 12, 978 (2022). 35454026 10.3390/diagnostics12040978 PMC9027801

[34] Jamshidi A. Evaluation of cell-free DNA approaches for multi-cancer early detection. Cancer Cell 40, 1537–1549 (2022). 36400018 10.1016/j.ccell.2022.10.022

[35] Zhang Z. Systematic calibration of epitranscriptomic maps using a synthetic modification-free RNA library. Nat. Methods 18, 1213–1222 (2021). 34594034 10.1038/s41592-021-01280-7

[36] Blanco S. Stem cell function and stress response are controlled by protein synthesis. Nature 534, 335–340 (2016). 27306184 10.1038/nature18282 PMC5040503

[37] Schumann U. Multiple links between 5-methylcytosine content of mRNA and translation. BMC Biol. 18, 40 (2020). 32293435 10.1186/s12915-020-00769-5 PMC7158060

[38] Zhang Y. 5-Methylcytosine (m5C) RNA modification controls the innate immune response to virus infection by regulating type I interferons. Proc. Natl Acad. Sci. USA 119, e2123338119 (2022). 36240321 10.1073/pnas.2123338119 PMC9586267

[39] Smallwood SA Single-cell genome-wide bisulfite sequencing for assessing epigenetic heterogeneity. Nat. Methods 11, 817–820 (2014). 25042786 10.1038/nmeth.3035 PMC4117646

[40] Clark SJ Genome-wide base-resolution mapping of DNA methylation in single cells using single-cell bisulfite sequencing (scBS-seq). Nat. Protoc 12, 534–547 (2017). 28182018 10.1038/nprot.2016.187

[41] Zhang Y, Park C, Bennett C, Thornton M & Kim D Rapid and accurate alignment of nucleotide conversion sequencing reads with HISAT-3N. Genome Res. 31, 1290–1295 (2021). 34103331 10.1101/gr.275193.120 PMC8256862

[42] Dai Q. Ultrafast bisulfite sequencing for efficient and accurate 5-methylcytosine detection in DNA and RNA. www.ncbi.nlm.nih.gov/geo/query/acc.cgi?&acc=GSE225614 (2023).

[43] Ye C. m5C-UBSseq. GitHub. github.com/y9c/m5C-UBSseq (2023).

