# Supplementary material for: Ultrafast bisulfite sequencing detection of 5-methylcytosine in DNA and RNA
Source: Nat Biotechnol. Author manuscript; Available in PMC 2025 Oct 1. (PMC11217147; doi:10.1038/s41587-023-02034-w)
Supplement: Sup Info [file NIHMS1990038-supplement-Sup_Info.pdf]

nature biotechnology

Article

https://doi.org/10.1038/s41587-023-02034-w

Ultrafast bisulfite sequencing detection of 5-methylcytosine in DNA and RNA

In the format provided by the authors and unedited

Supplemental item titles

Supplementary Table 1. Oligo and primer sequences used in this study.

Supplementary Table 2. Unconverted ratio of C sites on spike-in lambda DNA within libraries constructed from 1~100 cell(s) using both conventional BS-seq and UBS-seq.

Supplementary Table 3. Unconverted ratio of C sites on mtDNA within libraries constructed from 1~100 cell(s) using both conventional BS-seq and UBS-seq.

Supplementary Table 4. cfDNA methylation level of CpG sites within 10 kb slice window in libraries constructed with both conventional BS-seq and UBS-seq.

Supplementary Table 5. tRNA m^5C sites detected in small RNA libraries of WT A549 WT and NSUN2 knockout samples.

Supplementary Table 6. RNA m^5C sites detected in $polyA^+$ RNA libraries of WT HeLa samples.

Supplementary Table 7. RNA m^5C sites detected in $polyA^+$ RNA libraries of WT HEK293T samples.

Supplementary Table 8. RNA m^5C sites detected in $polyA^+$ RNA libraries of HeLa shControl and shNSUN2 samples.

Supplementary Table 9. RNA m^5C sites detected in $polyA^+$ RNA libraries of HeLa siControl and siNSUN6 samples.

Supplementary Table 10. Comparison of library quality statistics of UBS-seq with conventional BS-seq libraries.

Supplementary Note 1. High confident m5C sites calling by modeling the background noise.

Supplementary Data Tables (Legend)

Supplementary Table 1. Oligo and primer sequences used in this study.

Supplementary Table 2. Unconverted ratio of C sites on spike-in lambda DNA within libraries constructed from 1~100 cell(s) using both conventional BS-seq and UBS-seq.

Unconverted ratio of C sites on spike-in lambda of 3 replicates for libraries constructed from 1~100 cell(s) using both conventional BS-seq and UBS-seq are listed. Sites with less than 20× sequencing coverage were filtered. Column *'sample'* shows the name of the library; Column *'pos'* shows the relative position of the detected site to the reference sequence; Column *'strand'* shows the strand of detected site on the reference; Column *'unconverted'* shows the number of unconverted C on this site; Column *'converted'* shows the number of converted C on this site; Column *'ratio'* shows the 5mC stoichiometry.

Supplementary Table 3. Unconverted ratio of C sites on mtDNA within libraries constructed from 1~100 cell(s) using both conventional BS-seq and UBS-seq.

Unconverted ratio of C sites on mtDNA of 3 replicates for libraries constructed from 1~100 cell(s) using both conventional BS-seq and UBS-seq are listed. Sites with less than 20× sequencing coverage were filtered. The data structure of this table is similar to Supplementary Table 2.

Supplementary Table 4. cfDNA methylation level of CpG sites within 10 kb slice window in libraries constructed with both conventional BS-seq and UBS-seq.

The methylation level of CpG sites within a 10kb sliding window is combined. Two replicates for BS-seq and UBS-seq libraries are listed in the table. The sum of the numbers of converted or unconverted CpG sites within the sliding window is shown.

Supplementary Table 5. tRNA m^5C sites detected in small RNA libraries of WT A549 WT and NSUN2 knockout samples.

m^5C sites were detected in two replicates of WT and two replicates of *NUSN2* KO libraries of A549 cell line. Sites with no more than 3 coverages were discarded, and only sites with 5% of modifications in both replicates of WT libraries are listed in the table. Column *'gene'* shows the tRNA gene that the detected site is located; Column *'pos'* shows the relative position of the detected site to the tRNA sequence; Column *'WT-rep1'*, *'WT-rep2'*, *'NSUN2KO-rep1'*, *'NSUN2KO-rep2'* shows the m^5C stoichiometry in two replicates of WT and two replicates of *NSUN2* KO libraries respectively.

Supplementary Table 6. RNA m^5C sites detected in polyA$^+$ RNA libraries of WT HeLa samples.

m^5C sites that detected in three technical replicates of polyA$^+$ RNA libraries from HeLa cells. The total number of reads for each site across three libraries was then filtered using a p-value (binomial test) threshold of 10^{-6}. The mapping of rRNA / tRNA genes and the mapping of the genome are combined to produce a single list of loci. Column *'chrom'* shows the chromosome name of the site; Column *'position'* shows the position on the chromosome; Column *'strand'* shows the strand of detected site on the genome; Column *'gene_type'* shows the RNA species that the detected site is located, such as tRNA, lncRNA, and protein_coding; Column *'gene_name'* shows the gene that the detected site is located, with both Ensembl ID and common name are combined; Column *'gene_pos'* shows the relative position of the detected site to the gene; Column *'unconverted'* shows the number of unconverted C on this site; Column *'converted'* shows the number of converted C on this site; Column *'ratio'* shows the m^5C stoichiometry.

Supplementary Table 7. RNA m^5C sites detected in polyA$^+$ RNA libraries of WT HEK293T samples.

m^5C sites that detected in three technical replicates of polyA$^+$ RNA libraries m5C sites derived from HEK293T cells, with data structures similar to Supplementary Table 6.

Supplementary Table 8. RNA m^5C sites detected in polyA$^+$ RNA libraries of HeLa shControl and shNSUN2 samples.

m^5C sites detected in protein coding genes of HeLa cells are listed in the table. The data structure is similar to Supplementary Table 6, but 3 extra columns are shown. Column *'ratio_shControl'* shows the average m^5C stoichiometry in shControl libraries; Column *'ratio_shNSUN2'* shows the average m^5C stoichiometry in shControl libraries; Column *'delta_ratio'* shows the relative change of m^5C stoichiometry.

Supplementary Table 9. RNA m^5C sites detected in polyA$^+$ RNA libraries of HeLa siControl and siNSUN6 samples.

m^5C sites detected in protein coding genes of HeLa cells are listed in the table, with data structures similar to Supplementary Table 8.

Supplementary Table 10. Comparison of library quality statistics of UBS-seq with conventional BS-seq libraries.

Library quality statistics are presented for both low input mESC samples and single-cell mESC samples. Column '# PCR cycles' shows the number of PCR cycles used for sequencing libraries preparation; Column 'yeild (ng)' shows yield of libraries after PCR and purification; Column 'Fragment size' shows insert size of DNA fragments; Column 'Mapping ratio (%)' shows mapping ratio of clean reads to reference genome; Column 'Duplication ratio' shows the fraction of reads that were removed due to PCR duplicates; Column

'library size' shows effective library size; Column 'Genomic coverage' shows the proportion of the genomic region covered by the sequencing libraries.

Supplementary Note 1: High confident m^5C sites calling by modeling the background noise

The canonical analysis method for calling modified sites in RNA involves setting cutoffs based on the number of supporting reads (reads with mutation or deletion), the ratio of supporting reads, and sequencing coverage. However, this approach is arbitrary and lacks statistical power as it relies on finding the best combination of cutoffs through a subjective process.

The ratio of background noise (e) due to insufficient bisulfite conversion is assumed to be a constant value, which is typically around 0.002 under the optimized UBS condition. The sequencing coverage (d) of different sites in RNA libraries can vary greatly, ranging from just a few reads to several thousands. To detect RNA modification sites, a cutoff on the number of supporting reads is represented as "n", while a cutoff on the ratio of supporting reads is represented as "r". We can estimate the number of false positives under different detection cutoffs by simulating the random error following a binomial distribution, using formula (1).

$$P(X > n) = 1 - \sum_{i=0}^{\lfloor n \rfloor} \binom{d}{i} \mathbf{e}^i (1 - \mathbf{e})^{d-i}$$

Formula (1)

Our simulation revealed that applying a constant cutoff on the number of supporting reads (n) would be too stringent for sites on low expressed genes, but too loose for sites on high expressed genes. Conversely, applying a constant cutoff on the ratio of supporting reads would have the opposite effect, with sites on low expressed genes contributing more false positives to the filtering results (**Supplementary Note Fig. 1**).

It should be noted that even a modest 5-fold increase in background noise (from e=0.002 to e=0.01) can result in a significant increase in the false positive rate (FP%) by several orders of magnitude. For instance, when using a detection cutoff of 30 sequencing coverage and more than 3 unconverted reads, the FP% can increase by 529-fold by comparing the simulation in **Supplementary Note Fig. 1 and 2**.

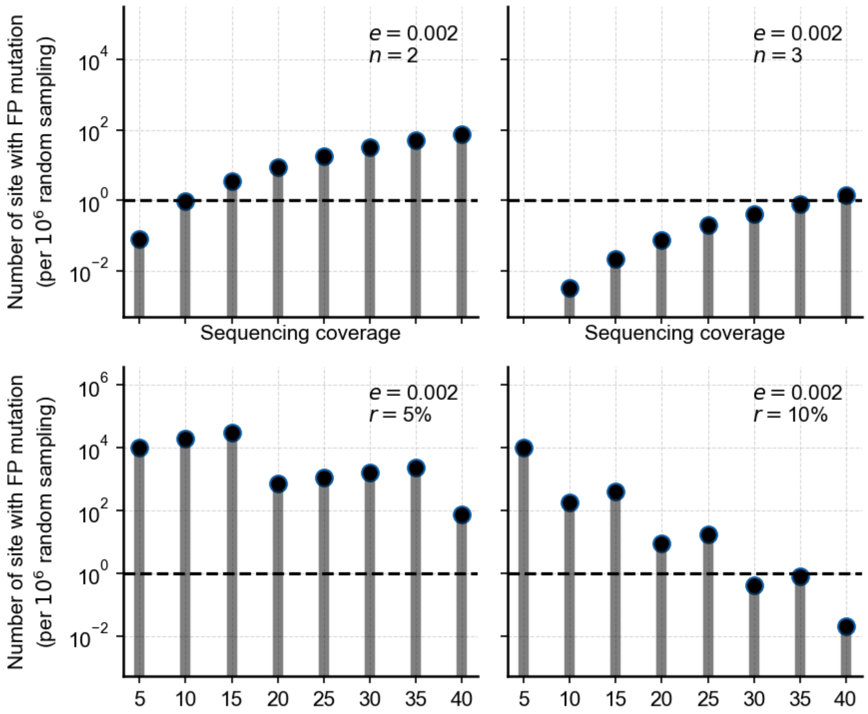

Supplementary Note Figure 1. Simulate number of false positives out of per 10^6 random sampling events when applying different cutoffs on either the number of supporting reads or the ratio of supporting reads. Background noise is set to **0.002**, which is typical for UBS-seq samples. When a cutoff of more than 2 unconverted reads (top left panel) was applied, the number of false positives can be as high as 100. In contrast, when a cutoff of more than 3 unconverted reads was applied (top right panel), most false positives can be removed, but this cutoff is too stringent for sites on low expressed genes and may result in some false negatives. When a constant cutoff of more than 5% of reads being unconverted was used (bottom left panel), sites with 5-40 sequencing coverage all show hundreds to thousands of false positives. When applying a constant cutoff of more than 10% of reads being unconverted (bottom right panel), sites on high expressed genes would show fewer false positives.

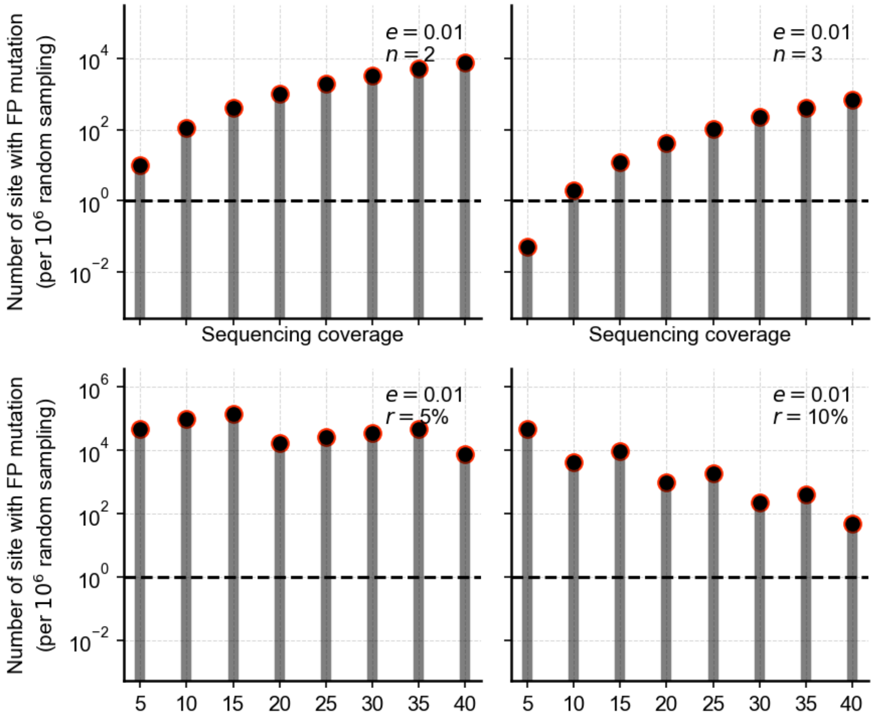

Supplementary Note Figure 2. Simulate number of false positives out of per 10^6 random sampling events when different cutoffs on either the number of supporting reads or the ratio of supporting reads were applied. Background noise is set to **0.01**, which is typical for conventional BS-seq samples. Applying the same cutoffs as shown in **Supplementary Note Fig. 1**, the number of false positives in all situations is 100 to1000-fold higher than using 0.002 as the background noise.

Due to the variability in sequencing coverage across RNA libraries, constant cutoffs cannot be used to accurately filter the background noise. To remove false positives completely, it is necessary to use different cutoffs for different sequencing coverages, which can account for the varying levels of noise and expression across sites. Formula (1) can be used to calculate a p-value, based on the number of unconverted reads, total sequencing coverage, and expected background noise. For different RNA libraries, the background noise can be calculated separately from all the C sites, most of which are expected to be unmodified and have very few unconverted reads.

As shown in **Supplementary Note Fig. 3**, the background noise observed in the UBS-seq samples used in this study is significantly less than 1%, which represents the best performance achievable using conventional BS-seq protocols.

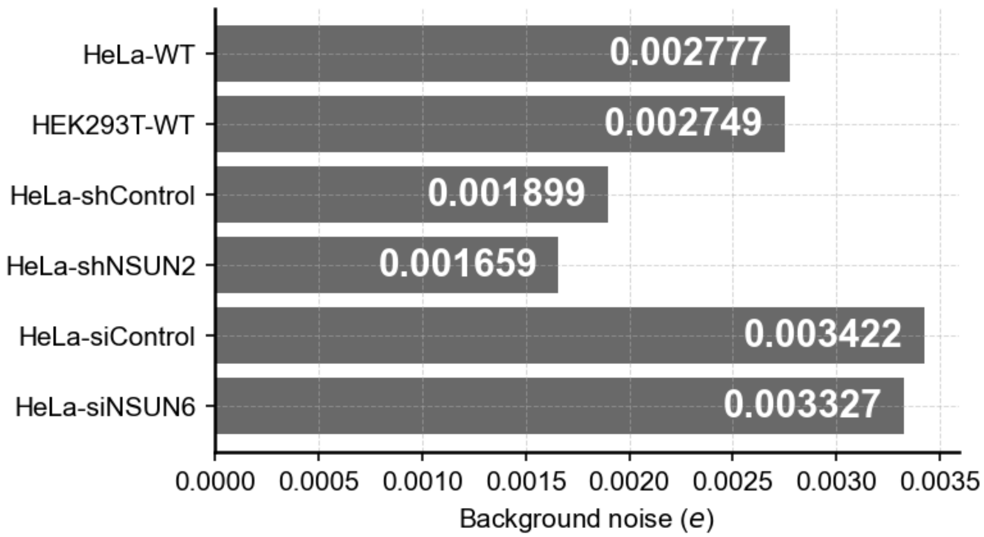

Supplementary Note Figure 3. Real background noise (e) of different UBS-seq libraries estimated from all the unconverted C sites in the sequencing data. The expectation, which is the average number, is labeled on the bar. It demonstrates that the background noise for UBS-seq samples used in this study is far less than 1%, which is the 5-10 times lower than conventional BS-seq protocol.

Using the true background noise for each library and the cutoff based on p-value, we can model the distribution of false positives among all the C sites (**Supplementary Note Fig. 4**). Then applying a threshold that p-value is less than 10^{-6}, we can detect high confident m^5C sites in the whole transcriptome.

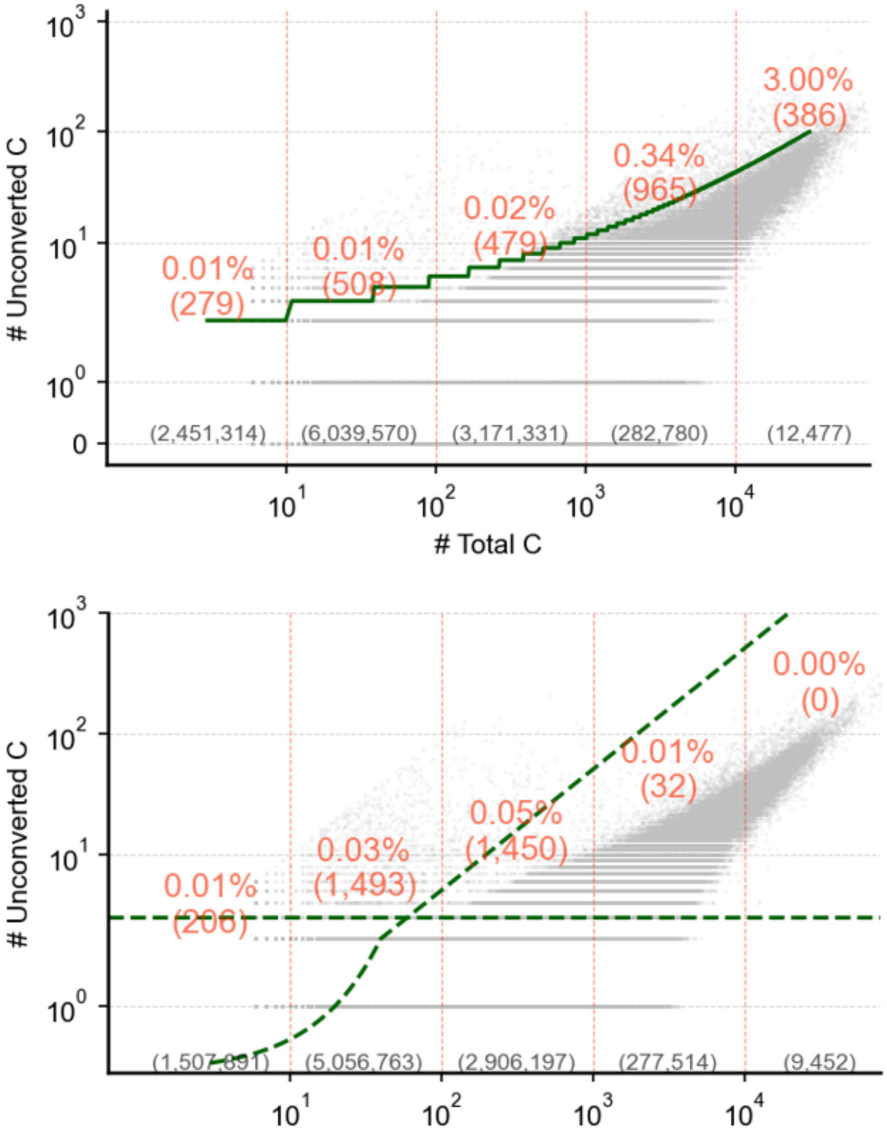

Supplementary Note Figure 4. Fitting the unconverted reads in background C sites in HeLa WT libraries using p-value based model and conventional model. A cutoff with p-value less than 10^{-6} (upper panel) can remove most of the false positives, and fit the real distribution of the data well, which could reduce false negatives in the detection as well. On the other hand, the conventional model (lower panel) with cutoffs of more than 3 unconverted reads and 2% of unconverted sites does not fit the distribution of the data well, and it also exhibits higher false negative ratios at sites on low expressed genes.

In addition, despite advancements in mapping software, accurately assigning reads to repeat sequences and homology loci remain challenging, particularly in the context of analyzing BS-seq data using 3-letter mapping. To minimize analysis errors, any site where 20% of mapping reads are found to be multiple-mapped will be filtered.

References

1. Huang, T., Chen, W., Liu, J., Gu, N. & Zhang, R. Genome-wide identification of mRNA 5-methylcytosine in mammals. *Nat Struct Mol Biol* **26**, 380–388 (2019).
2. Zhang, Z. *et al.* Systematic calibration of epitranscriptomic maps using a synthetic modification-free RNA library. *Nat Methods* **18**, 1213–1222 (2021).
3. Yang, X. *et al.* 5-methylcytosine promotes mRNA export - NSUN2 as the methyltransferase and ALYREF as an m5C reader. *Cell Res* **27**, 606–625 (2017).
